# Augmenting ultrasound for continuous glucose monitoring via a wearable acoustically readable microneedle patch

**DOI:** 10.1126/sciadv.aec3209

**Published:** 2026-05-01

**Authors:** Wanglinhan Zhang, Jiangang Xu, Xinjia Li, Yeji Jang, Qiqi Liu, Weixin Ma, Guojie Luo, Ming Ma, Qingqing Wang, Yi He, Qi Yuan, Jae-Woong Jeong, Long Meng, Zhongqing Su

**Affiliations:** ^1^ Department of Mechanical Engineering, The Hong Kong Polytechnic University, Kowloon, Hong Kong Special Administrative Region.; ^2^ School of Electrical Engineering, Korea Advanced Institute of Science and Technology, Daejeon 34141, Republic of Korea.; ^3^ State Key Laboratory of Biomedical Imaging Science and System, Shenzhen Institutes of Advanced Technology, Chinese Academy of Sciences, Shenzhen 518055, China.; ^4^ Department of Medical Ultrasonics, The Eighth Affiliated Hospital of Sun Yat-sen University, Shenzhen 518033, China.

## Abstract

Continuous glucose monitoring (CGM) represents substantial advancement, yet poses a challenge in wearable health care development. Current enzymatic CGM faces limitations in stability, cost, and durability. We introduce an enzyme-free, wearable acoustically readable microneedle patch (ARMPatch) composed of glucose-responsive hydrogel. Positioned between a standard ultrasound probe and the skin, ARMPatch acts as an acoustic interface enabling CGM using conventional ultrasound. Its hydrogel microneedles minimally penetrate the epidermis, allowing interstitial fluid to trigger variable swelling of the microneedles in response to glucose fluctuations. ARMPatch delivers stable and selective glucose readings for up to 56 days. Glucose variations with millimolar resolution are obtained via ultrasound within a response time of 30 to 60 minutes. In vivo CGM in animal models for 7 days reveals a reversible correlation between microneedle swelling and glucose variations. Acting as “accessory” for standard ultrasound probes, this approach offers a minimally invasive, cost-effective, and long-lasting solution to non-enzymatic CGM, expanding the utility of ultrasound in wearable biosensing.

## Teaser

An enzyme-free, wearable hydrogel microneedle patch empowers ultrasound to monitor blood glucose in a minimally invasive manner.

## INTRODUCTION

Diabetes, a common endocrine disease as evidenced by a sustained high blood glucose level, represents a critical global health challenge, accountable for the elevated rates of premature mortality and severe chronic complications (*1*, *2*). Regular glucose monitoring, or more ambitiously, continuous glucose monitoring (CGM), is imperative for early awareness of the abnormality in blood glucose level and subsequent medical intervention (*3*, *4*). For decades, fingerstick glucometers have been the primary means for calibrating blood glucose level—an invasive method giving rise to patients’ pain and inconvenience (*5*, *6*). Moreover, conventional glucometers are incapable of monitoring fluctuation in blood glucose level in a continuous manner. It is the recent advances in biochemistry and electronics that have propelled the rapid development of minimally invasive CGM techniques, some of which have been commercialized as consumer health-care products (*7*). Most commercial products in this category rely on enzyme-catalyzed reactions, but it is understood that enzymes are prone to restrictions such as short active life, high costs, and stringent storage requirements, hindering the widespread adoption of enzyme-based CGM techniques in clinics or daily health care (*8*–*10*).

Motivated by this, the past few years have witnessed intensive exploration of alternative CGM approaches without relying on enzyme-catalyzed reactions. These approaches use enzyme-free materials that are responsive to glucose, as typified by noble metals, transition metal oxides, and glucose-responsive hydrogels (*11*–*17*). In particular, the glucose-responsive hydrogel features specific functional groups binding to glucose molecules in physicochemical environments. Induced by this binding process, the hydrogel manifests a reversible volumetric change that can be correlated with fluctuation in glucose concentration (*17*–*20*). As a result of their high sensitivity to variation in glucose concentration, enzyme-free nature, superb biocompatibility, skin-like softness, and customizable material properties, the glucose-responsive hydrogel stands out among diverse enzyme-free materials and finds a wide spectrum of glucose-pertinent bioengineering applications, including diabetes wound treatment, insulin delivery, and enzyme-free CGM device development (*21*–*33*). At present, prevailing glucose-responsive hydrogel–based CGM techniques calibrate volumetric changes of hydrogels via optical measurement using dedicated and specialized instruments, which, however, could be excessively susceptible to ambient interferences such as background light or body movement (*28*–*35*). These hydrogels are often functionalized with chromogenic chemicals (e.g., fluorophores and colorimetric reagents), imposing a high risk of introducing toxicity to humans and losing functionality in the physiological milieu (*36*–*41*). There is an impending need to develop alternative methods that can circumvent the demerits associated with optical measurement and facilitate the implementation of enzyme-free CGM via glucose-responsive hydrogels.

Ultrasound continues to be a primary and predominant diagnostic tool for biomedical tissue imaging, detection of disease sources, and exclusion of pathologies (*42*, *43*). With the recent advances in micro-/nanomanufacturing, material sciences, and electronic packaging, targeted endeavor has been made to miniaturize bulky ultrasound equipment to a portable or even wearable scale, potentially expanding the use of ultrasound from clinical diagnosis by professionals in hospitals to regular health self-monitoring by patients at home (*44*–*54*). Studies have demonstrated that ultrasound-based calibration of hydrogel volumetric changes enables stable in vivo operation for over 24 days, while avoiding the ultraviolet (UV) or high-power laser exposure typically required for optical readout, and thereby improving both safety and long-term measurement stability (*55*–*60*). In addition, the volumetric change of hydrogels calibrated using conventional ultrasound probes carries physiological information. For instance, an ultrasound-readable bioresorbable hydrogel is developed to monitor homeostatic pH, which can further be used for the diagnosis of stomach leaks after surgery (*55*). A type of hydrogel sensor is injected into tissues for monitoring intracranial physiology using an ultrasound probe (*56*). These implantation- or injection-based monitoring strategies by ultrasound show prospects to realize CGM in a long-term and accurate fashion, although their invasive nature limits personalized applications (*61*). Therefore, the use of glucose-responsive hydrogels in conjunction with the ultrasound-based measurement appears to be an intriguing approach to realize enzyme-free CGM, while approaches of nonsurgical implementation remain absent.

In this study, we present an innovative approach to augment conventional ultrasound, to realize enzyme-free and minimally invasive CGM, via a wearable acoustically readable microneedle patch (ARMPatch). The patch is inserted between a standard ultrasound probe and the skin, serving as an acoustically augmentative interface between the probe and interstitial fluid (ISF). In a sense, the patch is analogous to an “accessory” for standard ultrasound probes. Fabricated with a phenylboronic acid (PBA)–based, glucose-responsive hydrogel, microneedles of the ARMPatch swell to respond to fluctuation in blood glucose level when the ARMPatch is applied to the skin. Hydrogel microneedles appear indistinguishable in ultrasound images, owing to the similar acoustic impedance with that of typical human tissue (*62*). To enhance the ultrasound contrast, biocompatible silica microspheres are embedded in hydrogel microneedles, enabling precise calibration of microneedle length changes in ultrasound images obtained via a standard ultrasound probe. Such acoustical modification endows the ARMPatch with the capability of converting the glucose level to a readable indicator in ultrasound images. The ARMPatch demonstrates a millimolar-level sensitivity to glucose fluctuations in a range from 0 to 40 mM as effective hyperglycemia alerts, a reversible response to simulated continuous changes in blood glucose level, and a stable calibration of glucose level for 56 days in vitro. To validate the proposed mechanism, we establish a chemical-mechanical coupling model to analyze the swelling behavior of hydrogel microneedles under the modulation of glucose concentration. With this model, the developed method and the ARMPatch can be easily modified to respond to other types of biomarkers. This study not only presents an unreported wearable device to boost conventional ultrasound for enzyme-free CGM, but also ushers an innovative avenue to monitor diverse physiological information via standard ultrasound in a minimally invasive manner using customizable hydrogel microneedles.

In contrast to the limited operational lifespan of enzyme-based CGM devices and the invasive procedures that are required by implantation-based CGM systems, our acoustically readable hydrogel microneedle platform offers a fundamentally different design paradigm. The ARMPatch enables potential operational stability in long-term use, while inducing only minimal invasiveness and requiring no professional medical intervention for application or removal. Moreover, ultrasound-based readout provides exceptional robustness against biochemical noise, as ultrasound propagates through tissue with negligible interference from endogenous molecular species, which is an intrinsic advantage over optical and electrochemical sensing modalities. As a result, our system demonstrates the feasibility of glucose monitoring in more than eight individual animals and 7 days of stable in vivo performance in a freely moving animal, highlighting its outstanding stability, safety, and translational potential relative to existing CGM approaches (table S1).

## RESULTS

### Concept, material, and fabrication of ARMPatch

**Fig. 1.**
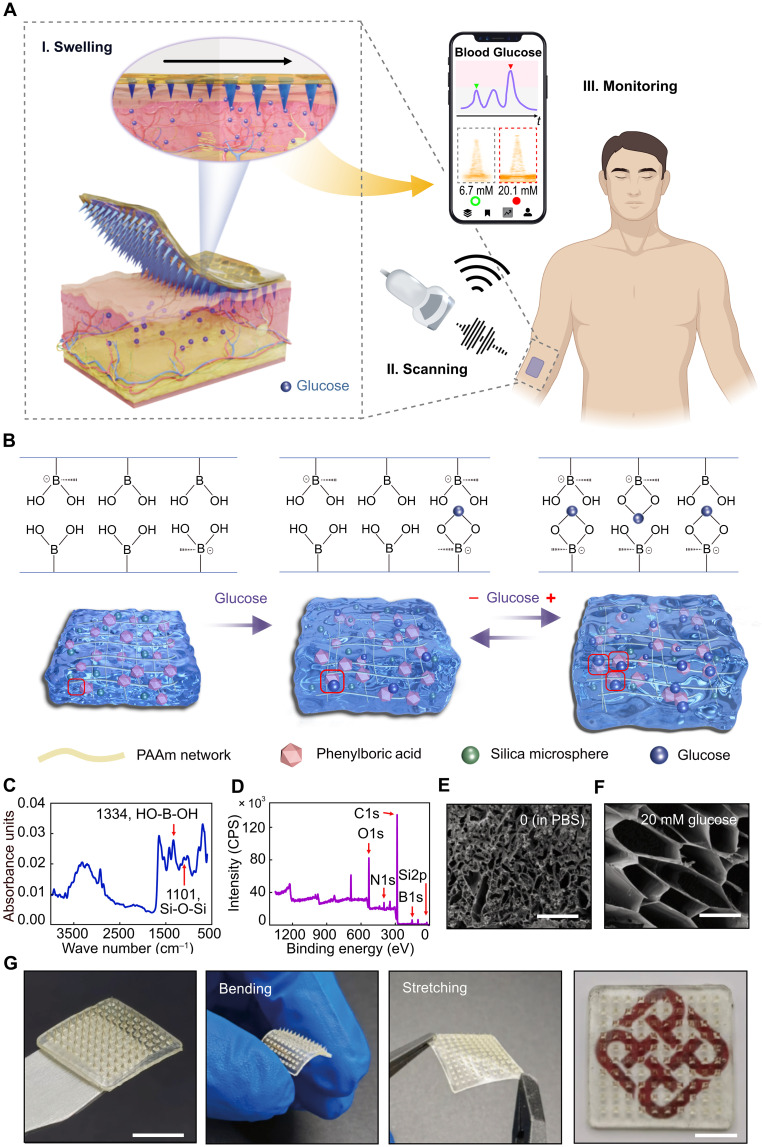
Concept, material, and fabrication of ARMPatch. (**A**) Schematic of the CGM configuration using a standard ultrasound probe in conjunction with the developed ARMPatch. The ultrasound image shown on the mobile phone is captured from the experiment in this study. Affinity Photo image editing software changes the lookup table (LUT) of colors in ultrasound images to obtain pseudocolor images. Created in BioRender. Jang, Y. (2026) https://BioRender.com/lt6ll33. (**B**) Schematic illustration of the mechanism of the glucose responsiveness of the synthesized hydrogel under the modulation of glucose. Chemical structures represent the materials used in this study. (**C**) FTIR analysis of the hydrogel sample to verify the presence of O─B─O and Si─O─Si. (**D**) XPS analysis of the hydrogel sample to verify the presence of B and Si. (**E**) Cross-sectional view (obtained via cryo-SEM) of the polymer network of the hydrogel without the treatment of glucose. Scale bar, 50 μm. (**F**) Cross-sectional view (obtained by cryo-SEM) of the polymer network of the hydrogel with the treatment of 20 mM glucose. Scale bar, 50 μm. (**G**) Optical images of a fabricated ARMPatch of 10-by-10 microneedle array, showing its flexibility under the bending and stretching states, respectively. Scale bars, 1 cm.

A schematic configuration of using the developed ARMPatch to augment a standard ultrasound probe and realize CGM is illustrated in Fig. 1A. The microneedles of the ARMPatch are made of a glucose-responsive hydrogel consisting of a polyacrylamide (PAAm) polymer network that is functionalized with PBA groups to form a specific glucose affinity. When applied to the skin, microneedles of the ARMPatch penetrate the epidermis to allow adequate interaction between the hydrogel and ISF beneath the epidermis. This penetration is of a minimally invasive manner, owing to the millimeter scale of the microneedles (~1 mm). The glucose molecules in ISF swell the hydrogel microneedles, and the degree of swelling changes in response to ISF glucose level. Given that the ISF glucose level correlates with that in the blood (*63*, *64*), the degree of microneedle swelling quantitatively reflects the blood glucose level. A standard medical ultrasound probe can simply be used to calibrate such a swelling degree via a standard ultrasound imaging process, in which the swelling is characterized by the length change of microneedles of the ARMPatch. To enhance ultrasound contrast of the hydrogel microneedles relative to surrounding tissue, silica microspheres are copolymerized within the hydrogel matrix as an acoustical modification, to increase the acoustic impedance of the hydrogel. Such a modification enables precision calibration of the length change of microneedles in ultrasound images. Consequently, the blood glucose level is “acoustically readable” in the images. To take a step further, with the popularity of wireless-enabled portable and wearable ultrasound devices, CGM for individuals with diabetes can be implemented by patients at home using these devices in conjunction with the ARMPatch. With this strategy, our design is expected to streamline and simplify the daunting task of accessing glucose information in physiological milieu using conventional ultrasound probes only.

The mechanism of the glucose responsiveness of the synthesized hydrogel is illuminated in Fig. 1B. Specifically, borate groups in PBA functional groups selectively bind to glucose molecules, generating a Donnan potential that increases osmotic pressure in the hydrogel matrix and leads to the swelling of the polymer network. As glucose concentration in physicochemical environment increases, the hydrogel manifests a varying degree of swelling, which is proportional to the glucose concentration (*32*, *39*). As this glucose-binding process is reversible, the hydrogel swells or deswells under the modulation of glucose fluctuation, making its volume change an indicator of the glucose concentration.

To substantiate the glucose responsiveness of the synthesized hydrogel with acoustical modification, we prepare a series of cubic samples (2 cm by 2 cm by 2 cm) of the synthesized hydrogel (detailed in Materials and Methods). Fourier transform infrared (FTIR) (Fig. 1C) and x-ray photoelectron spectroscopy (XPS) (Fig. 1D and fig. S1) analyses confirm the presence of PBA functional groups and silica microspheres in the synthesized hydrogel. As observed via scanning electron microscopy (SEM), the polymer network swells to a substantial scale in a physicochemical environment of 20 mM glucose, compared with a glucose-free environment (Fig. 1E and Fig. 1F), which affirms the glucose responsiveness of the synthesized hydrogel and the mechanism of glucose-modulated swelling. Details of the characterization are provided in Materials and Methods.

To further assess the glucose responsiveness of the hydrogel, eight of the prepared samples are immersed in glucose solutions of different concentrations, and their respective swelling degrees are determined as described in Materials and Methods. At a fixed sampling time, the swelling degrees of the samples exhibit a positive, stepwise increase from 1.5 to 11.3% (24 hours), along with the glucose concentrations in the range of 2 to 40 mM (fig. S2). In addition, the swelling degree of each sample continuously increases over time, for example, increasing from 4.4% at the time point of 3 hours to 12.3% at the time point of 48 hours (40 mM glucose), reflecting the intrinsic swelling characteristics of the synthesized hydrogel. This observation also accentuates the necessity of allowing an adequate response time for the hydrogel before the calibration of glucose concentration is conducted. This result highlights that the acoustically enhanced hydrogel retains glucose responsiveness, albeit with a reduced swelling degree (a 7.3% decline for 40 mM glucose within 24 hours), relative to the hydrogel without embedded silica microspheres (fig. S3). All results reveal the chemical composition of the synthesized hydrogel, confirm its glucose responsiveness with the acoustical modification, and verify its glucose-responsive mechanism.

Following the above material characterization and verification, a standard micromolding process (*65*) is adopted to fabricate the ARMPatch. The ARMPatch is an array of hydrogel microneedles, which are bonded to a light-cured flexible resin substrate. A silicone negative microneedle mold (fig. S4B) is obtained by demolding a copper positive mold (fig. S4A). The hydrogel monomer solution and the resin are sequentially cast in the silicone mold and cured by UV light, followed by a demolding process (fig. S5). Figure 1G presents a thus-fabricated ARMPatch with a 10-by-10 microneedle array. The scale, flexibility, stretchability, and transparency of the ARMPatch demonstrate its suitability for wearable applications. The device can be affixed to the skin using a medical tape or a biocompatible adhesive, and its flexible substrate enables conformal attachment, accommodating body movements without dislodgement. Details about the material and fabrication process are provided in Materials and Methods.

### Characteristics and functionalities of ARMPatch

**Fig. 2.**
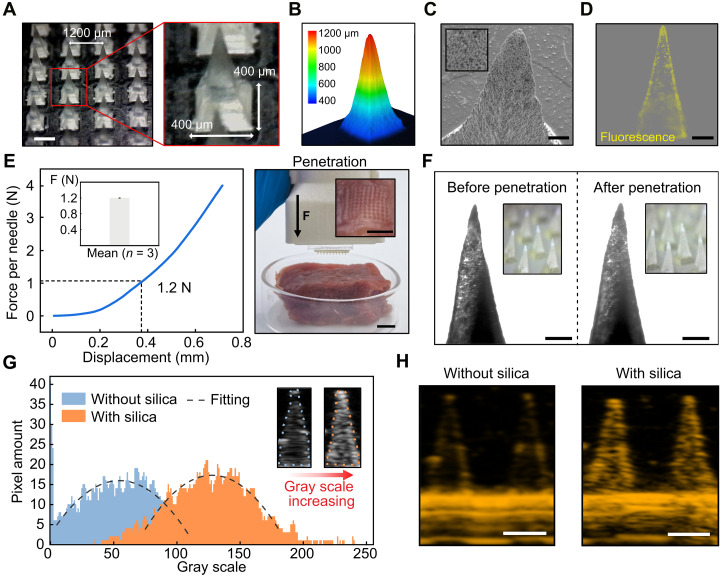
Characterization and functionalities of ARMPatch. (**A** to **D**) Characterization of the ARMPatch, including the size of the array (A), height of the microneedle (B), distribution of silica microspheres on the surface (C), and distribution of silica microspheres in the microneedle by fluorescence (D). Scale bars, 500 μm (A); 100 μm (C); 200 μm (D). (**E**) Mechanical strength of microneedles for penetration. Left: Force-displacement curve of a single microneedle in the compression test and the average result among three devices. The error bar refers to standard deviation (SD). Right: Puncture mark on porcine tissue after the ARMPatch being applied for 10 s. Scale bar, 1 cm. (**F**) Microscope images of a microneedle tip and the microneedle array before and after penetrating the porcine tissue. Scale bars, 200 μm. (**G**) Distribution of the gray scale value of microneedles with and without silica microspheres in ultrasound images, showing the distinct ultrasound contrast of the two compositions. Gray scale data in ultrasound images are derived from the intensity of ultrasound echoes ranging from 0 to 255, normalized by the imaging algorithm. (**H**) Comparison of ultrasound contrast of the microneedles with and without silica microspheres in ultrasound images (40 MHz; gain = 30; dynamic range = 65). Ultrasound images with pseudocolor are obtained by modifying LUT using the image editing software. Scale bars, 500 μm.

We characterize the fabricated ARMPatch via a series of morphological analyses. The optical microscopy image (Fig. 2A) highlights the size of the microneedle array with a tip-tip distance of 1200 μm and a base width of 400 μm. The laser confocal microscopy image (Fig. 2B) confirms the height of the microneedle of 1200 μm, which provides adequate resolution in ultrasound imaging for length calibration. The SEM image (Fig. 2C) depicts the distribution of silica microspheres on the surface of a microneedle. The fluorescence microscopy image (Fig. 2D) suggests the aggregation of silica microspheres at the microneedle tip—an acoustical modification that increases the ultrasound contrast of the tip and enables an accurate recognition of the tip in ultrasound images.

The mechanical strength of microneedles of the ARMPatch is evaluated via a standard compression test. When the displacement of the platen reaches 0.4 mm, which exceeds the depth required for full epidermal penetration, the average compression force applied on each microneedle is measured as 1.2 N, under which all microneedles are observed to remain their original integrity without any observable fracture (Fig. 2E, left). Tests on three different ARMPatches exhibit the consistently identical average compression force of 1.2 N at the displacement of 0.4 mm with a standard deviation of 0.012, indicating the stable performance across devices (inset of Fig. 2E, left). This demonstrates the adequate mechanical strength of the fabricated hydrogel microneedles—a critical factor to warrant a full epidermal penetration of the microneedles (*66*–*68*). This is further evidenced by the puncture mark observed on the porcine tissue after the patch is applied for 10 s (Fig. 2E, right). In addition, silica microspheres embedded in the hydrogel are observed not to adversely affect the mechanical strength of microneedles, as proven by the unchanged mechanical strength between microneedles with and without silica microspheres (fig. S6). As seen in Fig. 2F, the microneedles retain their original sharpness and aligned tips, before and after their penetration into the porcine tissue, indicating an adequate stiffness of the hydrogel microneedles to preserve the original pyramidal shape, benefiting accurate length calibration in ultrasound images. Such a feature effectively minimizes the calibration error induced by possible deformation of microneedles during epidermal penetration. Details of the setup are provided in fig. S7 and Materials and Methods.

To demonstrate the ultrasound contrast of the hydrogel microneedles enhanced by the embedded silica microspheres, we compare ultrasound images of two types of microneedles inserted into a skin phantom (1% agarose gel): hydrogel microneedles with the acoustical modification (with silica microspheres) and pristine hydrogel microneedles (without silica microspheres). In gray scale (0 ~ 255) ultrasound images, the microneedles with silica microspheres exhibit a higher gray scale value in the range of 80 ~ 180, revealing stronger ultrasound echoes, compared with the pristine microneedles without silica microspheres, which show a lower gray scale value in the range of 0 ~ 100 (Fig. 2G). This proves that the acoustical modification enhances ultrasound contrast of the barely discernible microneedles made of pristine hydrogel, in the presence of the background noise (Fig. 2H). To investigate the influence of the scanning angle of the ultrasound probe on the imaging, we compare the ultrasound contrast of microneedles with and without silica microspheres, obtained using a standard ultrasound probe moving along the skin phantom, as illustrated in movie S1. The movie suggests that microneedles with the acoustical modification facilitate the length calibration throughout the entire ultrasound scanning period, independent of the scanning angle. Thus, the acoustical modification is deemed necessary and effective. As previously demonstrated, this modification preserves both the glucose responsiveness and mechanical strength of microneedles, therefore holding application prospects of using the acoustically enhanced microneedles to extract different physiological information in ISF via regular ultrasound imaging. The experimental setup is illustrated in fig. S7 and Materials and Methods.

### CGM using ARMPatch: In vitro tests

With the proven glucose responsiveness of the synthesized hydrogel and functionalities of the microneedles, we evaluate the in vitro glucose-sensing performance of the ARMPatch. A series of identical ARMPatches are respectively immersed in glucose solutions of different concentrations, and we calibrate the change in the microneedle length through a standard imaging process using a commercial ultrasound probe. The swelling ratio (SR) of microneedles serves as an indicator of the length change, which is defined as (*l*_1_ − *l*_0_)/*l*_0_ where *l*_0_ is the initial microneedle length and *l*_1_ is the length upon change, real-time measured in an ultrasound image. All of the following SRs mentioned represent the average value of SRs of three microneedles that are randomly selected from each microneedle array (10 arrays in a fabricated ARMPatch), which are obtained simultaneously via the ultrasound imaging process under respective conditions.

**Fig. 3.**
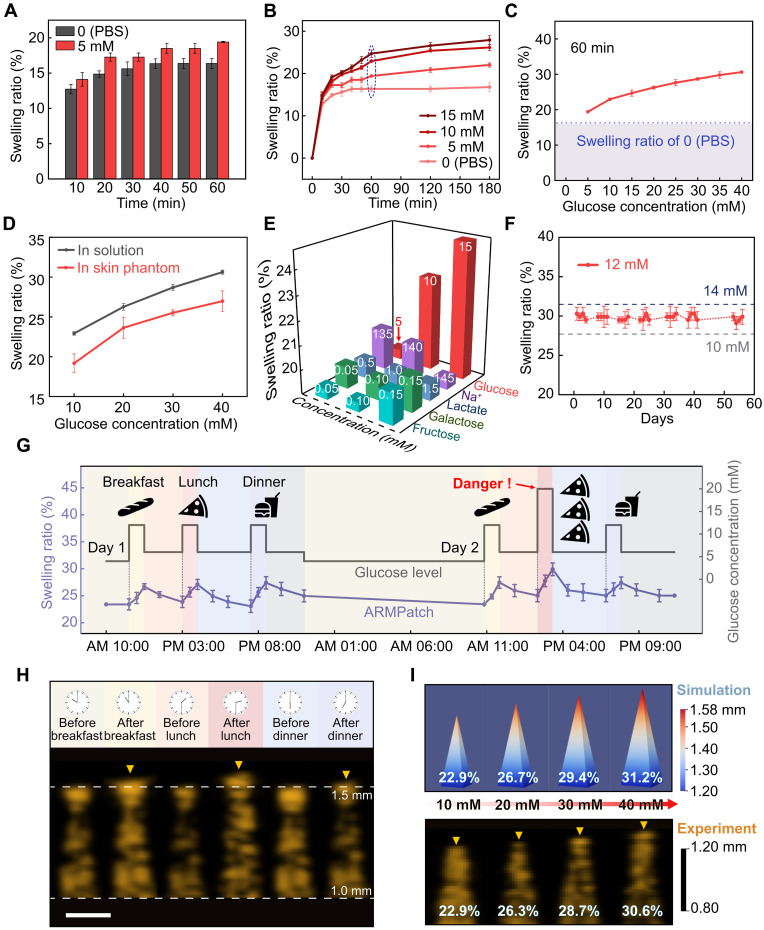
CGM using ARMPatch: In vitro tests. (**A**) Comparison of changes in SRs of the ARMPatches in a 5 mM glucose solution and a glucose-free solution within 60 min. (**B**) SRs of the ARMPatches changing over 180 min in the glucose solutions ranging from 0 to 15 mM. (**C**) Glucose-sensing performance of the ARMPatch against the glucose concentration ranging from 0 to 40 mM under a 60-min response time. The dotted line represents the SR under a glucose-free condition. (**D**) Comparison of SRs of the ARMPatches in a constrained (in skin phantom) and a quasi-free (in glucose solution) scenario varying with glucose concentrations. (**E**) Bioselectivity of the ARMPatch among fructose, galactose, lactate, and Na^+^. Numbers on the columns represent the actual concentration of millimolar. (**F**) Durable stability of the ARMPatch within 56 days. (**G**) Variations in the SR of the ARMPatch against the simulated fluctuations in the blood glucose level of a typical patient with diabetes. (**H**) Ultrasound images of a microneedle with varying lengths in response to the simulated fluctuations during continuous monitoring. All ultrasound images are captured from the same microneedle and resized in width with the same ratio. Scale bar, 200 μm (length only). (**I**) Comparison of the model-predicted and experimental-measured SRs in response to different glucose concentrations (quasi-free swelling). Ultrasound images (40 MHz; gain = 30; dynamic range = 65) are selected from the microneedles with similar initial lengths after enlarging with the same ratio. All error bars refer to SD, *n* = 3. Ultrasound images with pseudocolor [(H) and (I)] are achieved by modifying LUT using the image editing software.

As representative results, SRs of two identical ARMPatches in response to a glucose solution of 5 mM and a glucose-free solution over time are compared in Fig. 3A. Within a measurement window of 10 min, the SR of the ARMPatch in the glucose solution rises to 14.1%, whereas that in the glucose-free solution rises to 12.7%, suggesting that the ARMPatch can detect changes in glucose concentration within a brief period. To determine the response time of the ARMPatch, we monitor the swelling of microneedles and continuously calibrate the corresponding SRs in response to different glucose concentrations (0, 5, 10, and 15 mM) over a window of 180 min (Fig. 3B). After 30 min, it is feasible to distinguish the glucose concentrations using the calibrated SRs, although the accuracy and resolution remain limited due to incomplete swelling of hydrogel microneedles. These SRs tend to stabilize within 60 min with an improved sensitivity to different glucose concentrations. In practical applications, possible delays of measurement may arise from multiple factors, including blood glucose elevation (~10 min), ISF diffusion (~20 min), and hydrogel swelling (>30 min), which occur concurrently. A longer response window, therefore, not only allows the hydrogel to approach a quasi-saturated swelling state for a more accurate readout of glucose concentration, but also integrates the combined effects of these physiological delays, enabling the ARMPatch to capture the comprehensive postprandial glucose peak rather than values from the intermediate rising or diffusing phase. Clinical studies further indicate that postprandial glucose peaks in patients with diabetes typically persist for 60 to 90 min, suggesting that a 60-min response time is appropriate and not excessively long (*69*, *70*). Therefore, while ultrasound-calibrated SRs after 30 min are sufficient to reflect glucose trends, a 60-min readout window provides a more accurate representation of the actual glucose level. This response time incorporates both the optimal swelling kinetics of the hydrogel and the characteristic postprandial glucose profile observed in patients with diabetes in clinics. Although a delay of 30 to 60 min may limit real-time monitoring of blood glucose level, clinical CGM systems primarily emphasize trend tracking over defined periods, such as postprandial excursions, fasting glucose patterns, and insulin resistance, rather than instantaneous values (*71*, *72*). Accordingly, despite the inherent delay, evaluating the SR of the ARMPatch over time remains meaningful for diabetic management, as it reflects long-term trends, and the response time can be further shortened through the optimization of the ARMPatch design (discussed in section S1). Based on these considerations, the calibration of the SR in the subsequent in vitro and in vivo tests adopts the SR obtained 60 min after patch application.

The glucose-sensing performance of the ARMPatch is investigated by immersing nine identical ARMPatches in glucose solutions of different concentrations and calibrating the SRs of respective ARMPatches with a standard ultrasound probe, as detailed in Materials and Methods. As observed, SRs of the ARMPatches stepwise increase from 19.4 to 30.6%, respectively, when the concentrations of glucose solutions range from 5 to 40 mM, proving a direct correlation between the SR calibrated by the ultrasound probe and the value of the glucose level (Fig. 3C). Within the typical physiological range of 0 to 20 mM, the sensitivity of the ARMPatch to changes in glucose concentration is ~0.5%/mM, as determined from the slope of the fitted regression line (fig. S8A). At higher glucose levels, the sensitivity is noted to decrease (fig. S8B), which is attributable to the limited boronate binding sites in the hydrogel matrix (*39*). Given that the adopted ultrasound imaging process resolves a minimum of 1% change in SR (i.e., 0.015 mm per pixel, corresponding to a change of 1.2 mm of a microneedle), the detection limit of the ARMPatch with the current experimental configuration is 2 mM for the typical physiological range. Having said that, such a detection limit is adequate for triggering a warning signal against severe hyperglycemia in CGM. By leveraging advances in ultrasound imaging resolution and optimizing hydrogel composition such as the cross-linking density and silica microsphere content, the detection limit of the patch can further be improved.

It is understood that when microneedles of the ARMPatch penetrate the epidermis, the surrounding tissue constrains the free swelling of the hydrogel and accordingly affects the microneedle length calibration. To investigate such an effect, we compare SRs of ARMPatches inserted in skin phantoms (1% agarose gel) of different glucose concentrations, a constrained scenario, and ARMPatches immersed in glucose solutions of different concentrations, a quasi-free scenario by ignoring the hydrostatic pressure of the solution (Fig. 3D). The detailed experimental setup is provided in Materials and Methods. In the constrained scenario (in skin phantoms), the sensitivity (i.e., the slope of the linear fitting) of the ARMPatch to the glucose variation is 0.252%/mM, which is close to the sensitivity of 0.255%/mM determined when the ARMPatch is under the quasi-free condition (in glucose solutions). Thus, it is safe to draw a conclusion that while the hydrogel microneedles tend to have a slight reduction (e.g., 3.6% at 40 mM) in the measured SR due to the tissue constraints, the sensitivity of the ARMPatch to glucose variation remains unaffected. This way, calibration of the glucose level may vary between individuals due to physiological differences, but it remains consistent for a particular patient, benefiting the reliability of CGM.

Previous studies (*14*, *32*, *39*) indicate that the PBA functional group binds not only to glucose but also to other diol-containing saccharides such as galactose and α-hydroxy acids such as lactic acid. In addition, ions (e.g., Na^+^) in ISF also alter the swelling degree of the hydrogel and accordingly influence the length of microneedles. We investigate the bioselectivity of the ARMPatch against four different substances in ISF that may interfere with the response of the PBA functional group, including fructose, galactose, Na^+^, and lactate. Solutions of 5 mM glucose are prepared, each containing one of the four substances at its normal physiological concentration. Three different concentrations of glucose solutions (i.e., 5, 10, and 15 mM) are made as the control group. As observed in Fig. 3E, the change in glucose concentration leads to a maximum 5.2% increase of the SR in the control group (in the range of 5 to 15 mM), and in the meantime, the contribution to the variation in SRs from the four substances is less than 0.7% on average (Fig. 3E and fig. S9). These results validate that the possible interferences from typical substances in ISF (namely fructose, galactose, Na^+^, and lactate) within normal physiological ranges are negligible for the ARMPatch for CGM, proving the bioselectivity of the ARMPatch to glucose.

To assess the long-term glucose-sensing stability of the ARMPatch, a device is subject to alternating cycles of a glucose-free solution and a 12 mM glucose solution for 56 days. Details about the experiment are provided in Materials and Methods. The ARMPatch shows an average SR of ~29.3% (±0.7%) in response to a glucose concentration change from 0 to 12 mM in 56 days of experiment (Fig. 3F). After 56 days, the ARMPatch is still capable of differentiating between higher and lower glucose concentrations by the SR, as evidenced by presenting the SR of 30.4% for 14 mM and 28.0% for 10 mM. This demonstrates that the device is capable of monitoring the fluctuation in glucose level over an extended period. Unlike those CGM devices that use enzymes and require frequent replacement, the ARMPatch achieves superb operational life owing to its enzyme-free nature.

For continuous monitoring of blood glucose level, an ARMPatch is subject to solutions of different glucose concentrations, mimicking the daily ingestion-related variation in blood glucose level of a typical patient with diabetes. The detailed experimental setup is provided in Materials and Methods. Across a 2-day test, the SR of the ARMPatch rises from the initial 23.4% at the fasting level (4 mM) to 27.2% upon averaging the SR obtained from each measurement when the postprandial level (12 mM) is applied, and it then declines to a mean value of 24.5% when the postabsorptive level (6 mM) is applied (Fig. 3G). Notably, at those high glucose concentrations (i.e., 12 mM at postprandial level), the ARMPatch shows consistent reading of the SR across cycles with an error of 0.3% only, indicating a reliable calibration of the peak postprandial level by the ARMPatch. After the ARMPatch is immersed overnight in a solution of 4 mM glucose, which mimics the fasting level of the next day, the SR returns precisely to 23.4%, the same as the SR representing the fasting level of the previous day, proving an accurate return of the reading in each measurement cycle and irrelevance to SRs in previous measurement cycles. When the blood glucose level reaches a hyperglycemic level (20 mM), the SR rises to 29.9%, indicating the necessity for the users to take preventative treatment such as insulin injections. Close-up ultrasound images of the microneedles highlight the length change of the same microneedle (Fig. 3H), revealing evident and reversible responses of the ARMPatch that are read by ultrasound to variations in glucose concentration in a daily cycle. The results prove that the ARMPatch is a glucose-responsive and enzyme-free device that is capable of continuously monitoring blood glucose level and offering a hyperglycemia alert.

Although the swelling kinetics of hydrogels have been extensively investigated (*73*, *74*), a theoretical model that can depict the swelling of the fabricated hydrogel microneedles responding to the fluctuation in the glucose concentration in a physicochemical environment is still lacking. Here, we formulate a chemical-mechanical coupling model of hydrogel microneedles that accounts for the selective binding of glucose, the force equilibrium, and the solvent transport through the swelling process. The model is implemented via a finite element method to predict SRs of microneedles in response to different glucose levels in quasi-free and constrained scenarios. SRs of microneedles under the quasi-free condition predicted by the model are 22.9, 26.7, 29.4, and 31.2% when the glucose concentrations are 10, 20, 30, and 40 mM, respectively, which closely match the experimentally obtained values of 22.9, 26.3, 28.7, and 30.6% in the in vitro tests (Fig. 3I and fig. S10A). Simulation results of SRs for the time-dependent swelling of microneedles and the swelling of microneedles in the constrained scenario also agree with experimental results (fig. S10). As swelling is a generic response of hydrogels to external stimuli in a physicochemical environment, the hydrogel of the ARMPatch can further be tailor-made by modifying its composition, for responding to other types of biomarkers (e.g., pH or protein). Representatively, the glucose-binding parameter in our model here is generalized as a chemical variable for use in other stimulus-responsive hydrogels, while the parameter can be customized to reflect a diverse range of physiological information. Details are available in section S2.

### Proof-of-concept demonstration: In vivo tests

**Fig. 4.**
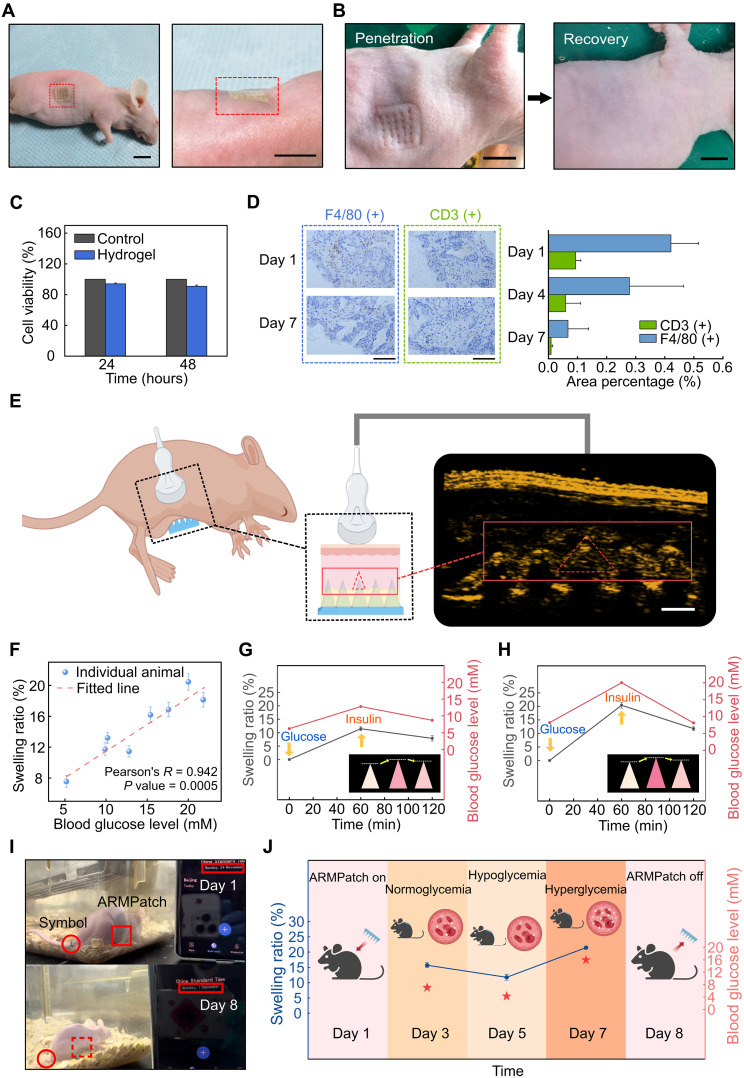
Proof-of-concept demonstration with animal models. (**A**) Optical images of the ARMPatch applied to the nude mouse obtained using a camera. Scale bars, 1 cm. (**B**) Optical image of the puncture mark induced by the ARMPatch on the skin and the recovery of the skin within 2 hours after the removal of the patch. Scale bars, 1 cm. (**C**) Cytotoxicity of the hydrogel within 48 hours. The blank control group represents a nonhydrogel condition for standard comparison. (**D**) F4/80 and CD3 staining results of the tissue after the ARMPatch was applied. Left insets: Selected images of tissue sections under a microscope. Scale bars, 50 μm. (**E**) Animal experiment configuration of glucose monitoring using the ARMPatch via ultrasound and the ultrasound image of the ARMPatch captured in vivo (40 MHz; gain = 60; dynamic range = 15). Scale bar, 1 mm. Created by figdraw.com. Copyright code: IAYOO3ab0b. (**F**) SRs of microneedles under different blood glucose concentrations obtained via ultrasound imaging in vivo and the validation of the linear relationship between the two parameters (sample size = 8). (**G**) Changes in SR of the ARMPatch calibrated from ultrasound images against a blood glucose level shifting in vivo. (**H**) Changes in SR of the ARMPatch calibrated from ultrasound images against another blood glucose level shifting in vivo. (**I**) Optical images of the mouse before and after a 7-day experiment of wearing the ARMPatch for CGM. (**J**) SRs of the ARMPatch calibrated from ultrasound images against the blood glucose levels of the mouse over 7 days. Created in BioRender. Jang, Y. (2026) https://BioRender.com/kucdukf. All error bars represent SD, *n* = 5 for (C) and *n* = 3 for others. Ultrasound images with pseudocolor in (E) are achieved by modifying LUT using the image editing software.

We conduct in vivo experiments using nude mice models to evaluate the performance of the ARMPatch for CGM. The setup of applying the ARMPatch to mice is elucidated in Materials and Methods. Optical images show that the ARMPatch is conformally applied to the mouse skin (Fig. 4A). Upon insertion into the epidermis, the patch leaves an observable puncture mark on the mouse skin (Fig. 4B, left), confirming adequate penetration of microneedles of the ARMPatch in the skin. In addition, the mark on the skin completely vanishes within 2 hours upon removal of the ARMPatch, accentuating a minimally invasive attribute of the device (Fig. 4B, right).

The biocompatibility of the ARMPatch is further validated through cytotoxicity and immunopathology assays. With the presence of the hydrogels, the cell viability remains above 90% within 48 hours (Fig. 4C), affirming the reliability and biosafety of the acoustically enhanced hydrogel. Immunopathology analysis of the tissue at the ARMPatch insertion site reveals only minimal macrophage and T lymphocyte infiltration, with the mean positive staining area remaining below 0.5% of the total tissue section, indicating a mild and localized immune response (*75*, *76*) that fully resolves within 7 days (Fig. 4D and fig. S11). In addition to the cytocompatibility of the hydrogel and minimal immune response of the skin, the hydrogel microneedles exhibit excellent long-term chemical stability. PAAm-based networks have a carbon-carbon backbone that is highly resistant to hydrolysis and enzymatic cleavage under physiological conditions, making the materials essentially nondegradable and insoluble. To experimentally verify this stability, a hydrogel sample is immersed in pure water for 7 days, followed by inductively coupled plasma mass spectrometry (ICP-MS) and total organic carbon (TOC) analyses to quantify the elements that existed in the solution. No detectable increase in silicon, boron, carbon, or nitrogen is observed, confirming that neither the embedded silica microspheres nor the hydrogel polymer chains leach or degrade over time (table S2). As for the safety of ultrasound exposure, there is no evidence of deleterious biological effects under standard diagnostic imaging conditions (*42*, *43*), and ARMPatch-based glucose monitoring involves only short and periodic ultrasound acquisitions, which further reduces any theoretical safety concerns. In conclusion, by leveraging the biocompatibility and stability of the hydrogel, the minimally invasive nature of the microneedles and the biosafety of ultrasound, the ARMPatch can function as a wearable device. Details about the biocompatibility tests are provided in Materials and Methods.

With verified biocompatibility, we investigate the ultrasound contrast of microneedles in vivo using a standard ultrasound probe. In the imaging setup (Fig. 4E), the mouse is positioned laterally, with one flank facing upward. The ultrasound probe is placed above the exposed abdominal surface to acquire vertical cross-sectional images. An ARMPatch is inserted contralaterally, from the lower side of the body, with its microneedles penetrating upward into the tissue. This orientation minimizes signal distortion that is caused by reflections of ultrasound from the ARMPatch substrate and therefore facilitates accurate calibration of the microneedle length. In the specific geometry shown in Fig. 4E, the cross-sectional view of the microneedles appears as high-contrast triangular shapes in the image, clearly distinguishable from background features associated with tissue. With the acoustical modification, microneedle tips are distinctly highlighted in the images, facilitating calibration of both the orientation and length of microneedles. Tests of the same configuration on different mice and insertion sites of the ARMPatch on the mouse skin are implemented, consistently yielding reliable ultrasound imaging results (fig. S12). For blank control comparison, when an ARMPatch is applied on the mouse skin with sufficient contact yet without penetrating the skin, the microneedle tips are not observable in the obtained images (fig. S13A). In a wide-view image of the internal cavity of the mouse, the microneedles are observable only in the region where the ARMPatch is applied (fig. S13B). This confirms that the observable microneedles can be safely attributable to the ARMPatch. In dynamic imaging (movie S2), certain hyperechoic regions exhibit synchronous motion with the cardiac cycle of the mouse, indicating dynamic displacement of the tissue associated with the animal’s heartbeats. In contrast, the microneedles and their tips remain stationary in the movie, confirming that these high-contrast geometries correspond to the microneedles of the ARMPatch rather than any part of the tissue of the mouse. Thus, microneedles with the acoustical modification are clearly visualized in vivo via ultrasound imaging, demonstrating that the ARMPatch enables accurate calibration of the SR and makes the glucose information “acoustically readable.” These findings underscore the importance of the acoustical modification in enhancing the ultrasound contrast of hydrogel, thereby enabling effective characterization of hydrogel microneedles with conventional ultrasound imaging only.

Before measuring blood glucose level of the mice using the ARMPatch via ultrasound imaging, we first use a microscope to confirm the SRs of three identical ARMPatches in response to different blood glucose levels of three mice, serving as a standard comparison. Different blood glucose levels in the mice are achieved by injecting glucose solutions of different gradient volumes to each mouse. Simultaneously, a commercial glucose meter is used to measure the blood glucose levels of all three mice for reference. The detailed experimental setup is provided in Materials and Methods. Using the microscope, the SRs of the ARMPatches are obtained as 10.3, 13.1, and 16.8%, respectively, for three mice, comparing the actual blood glucose levels of 7.8, 12.2, and 22.1 mM determined by the commercial glucose meter (fig. S14). These results verify that the glucose-sensing capability of the ARMPatch, previously proven in vitro, is retained in vivo.

Subsequently, eight ARMPatches are respectively applied to eight nude mice with different blood glucose levels, and ultrasound images are acquired using a standard ultrasound probe. Modulation of blood glucose level in mice follows the same protocol described above. The initial state of microneedles for each ARMPatch is imaged by an ultrasound probe immediately after the insertion of the patch, and the imaging process is repeated 1 hour after the glucose injection, to calibrate the SRs of the ARMPatch. The standard blood glucose levels are measured using a commercial glucose meter for comparison (see Materials and Methods). After calibration, the ARMPatches exhibit SRs of 7.6, 11.7, 13.2, 11.5, 16.2, 16.9, 20.4, and 18.1%, corresponding to blood glucose levels of 5.2, 9.9, 10.2, 12.8, 15.4, 17.6, 20.0, and 21.8 mM, respectively (Fig. 4F). Across all eight mice, the ultrasound-obtained SRs show a strong linear correlation with blood glucose levels measured by the commercial glucose meter (Pearson’s *R* = 0.942). Statistical analysis further confirms the robustness of this correlation, with a two-tailed *P* value of 0.0005, indicating high reproducibility and significance (detailed in section S3). These results demonstrate that ultrasound imaging effectively quantifies the SR of the ARMPatch in vivo within 1 hour, enabling accurate assessment of blood glucose fluctuations.

After verifying that the ARMPatch, in conjunction with the use of conventional ultrasound imaging, can accurately reflect blood glucose levels in vivo, we evaluate the capability of the device for CGM using ultrasound. This is achieved by producing cyclic changes of blood glucose level in the mice, through alternating injections of glucose and insulin in each cycle. The detailed experimental setup is provided in Materials and Methods. As illustrated in Fig. 4G, an ARMPatch applied to a mouse initially presents an SR of 11.5% in response to a blood glucose level of 12.8 mM following glucose injection, and the SR subsequently decreases to 7.9% when the blood glucose level drops to 8.7 mM after insulin administration, indicating the in vivo CGM capability of the device. In another trial of a different glucose level, an ARMPatch obtains an SR of 20.4% at a blood glucose level of 20.0 mM in a mouse after glucose injection, and the SR then declines to 11.8% at a glucose level of 8.1 mM in the mouse after insulin administration (Fig. 4H), further validating the reproducibility of this ultrasound-based CGM technique under different glucose levels. These in vivo findings reveal the practicability of augmenting conventional ultrasound for enzyme-free and minimally invasive CGM using the developed ARMPatch—an innovative approach that is fundamentally distinct from all conventional CGM methods.

Following the demonstration of the fundamental CGM ability of the ARMPatch, we investigate the long-term in vivo utility of this ultrasound-readout approach. Specifically, a nude mouse is prepared, and an ARMPatch is applied and secured on the mouse skin using a tissue adhesive. The ARMPatch is protected by a roll of tape that is removed during ultrasound imaging. The mouse is awake and free with the ARMPatch applied throughout the 7-day experiment (except for the short periods of ultrasound imaging). The blood glucose level of the mouse is modulated on day 3, day 5, and day 7 by applying no pretreatment, insulin injection, or glucose injection, respectively, to induce normoglycemic, hypoglycemic, and hyperglycemic conditions for testing. The mouse remains healthy, active, and responsive while wearing the ARMPatch throughout the entire experimental period (Fig. 4I and movie S3). Despite the mouse being able to move, feed, and rest freely, the ARMPatch stays securely attached without detachment or displacement for 7 days. The ultrasound-calibrated SR of the ARMPatch exhibits 15.7, 11.7, and 21.5% obtained on day 3, day 5, and day 7, respectively, matching the blood glucose level of 7.2, 4.4, and 16.1 mM measured by a commercial glucose meter, confirming the feasibility of using ultrasound-based microneedle length monitoring for long-term continuous glucose assessment (Fig. 4J). The microneedles maintain consistent readability in ultrasound imaging over the testing period, unaffected by the mouse’s movements. The skin of the mouse recovers without puncture marks by the following day after the removal of the ARMPatch and exhibits no signs of inflammation (Fig. 4I). The mouse is observed to be healthy after the experiment with normal behavior. These results demonstrate the biocompatibility, safety, and minimally invasive nature of the ARMPatch during prolonged use, establishing this ultrasound-enabled platform as a stable and effective way for CGM.

## DISCUSSION

While the glucose-responsive hydrogels render a promising solution to realize enzyme-free CGM, there remains a vital need for a physiologically stable method to extract glucose-related information from hydrogels without relying on complex and customized equipment. Addressing this challenge, ultrasound has adequately demonstrated its eminent capability to reliably and safely characterize hydrogels in vivo, although its current use remains largely confined to invasive clinical applications. In addition, the ongoing efforts toward portable and wearable ultrasound devices suggest the vast potential of conventional ultrasound in home health-care applications. Given these considerations, integrating ultrasound with glucose-responsive hydrogels to implement CGM is an attractive approach, particularly with the prospect of using portable and wearable ultrasound devices. That said, the development of a truly nonsurgical method remains essential.

To address this need, we developed the ARMPatch—a microneedle platform that integrates silica microspheres with a glucose-responsive hydrogel to create an acoustically readable interface for conventional ultrasound. The embedded silica microspheres provide robust acoustic contrast, enabling microneedle swelling to be directly quantified in ultrasound images and effectively decoupling the readout from biochemical noise and enzymatic instability. This design transforms blood glucose levels into a minimally invasive and readable indicator that can be captured using standard clinical or portable ultrasound probes. By integrating ultrasound, a safe and widely adopted imaging modality, with a biocompatible microneedle interface, the ARMPatch maximizes overall safety and biocompatibility for long-term physiological monitoring. Given that a large population of patients with diabetes have already used or are considering using portable or wearable ultrasound devices for monitoring complications such as cardiopathy and nephropathy, the ARMPatch offers a practical pathway to achieving CGM without requiring additional customized hardware. This developed platform has been validated in vitro and in vivo using nude mice models, demonstrating its wearability, glucose-sensing capability, and ability to operate continuously.

Beyond glucose monitoring, the concept of ARMPatch introduces additional innovation: It establishes a generalizable strategy for augmenting ultrasound with chemically responsive hydrogels to access diverse biomarkers in ISF. By modifying the hydrogel composition of the microneedles, the platform can, in principle, be adapted to respond to pH, proteins, bacteria, and other physiologically relevant analytes, thereby equipping portable and wearable ultrasound devices with distinct sensing capabilities that extend far beyond traditional anatomical imaging. To support this vision, we further propose and validate a chemical-mechanical coupling model that interprets microneedle length variation as a function of analyte concentration, and we evaluate the necessity of acoustical contrast enhancement as well as identify opportunities for future optimization. Together, these advancements highlight the ARMPatch as a unique and enabling interface that expands the functional landscape of ultrasound technology and opens emerging avenues for its application in personalized and at-home health care.

A practical challenge for future clinical translation of the proposed method lies in ensuring robust functionalities of hydrogel microneedles and ultrasound readout. Specifically, mechanical fatigue, tissue interactions, and potential biofouling may influence hydrogel swelling and acoustic readability. Our results indicate that the hydrogel response is largely robust under physiological mechanical stress, and the PBA-based glucose recognition is generally insensitive to nonspecific bioadsorption, supporting a stable operational window compared with enzyme-based systems. In contrast, the imaging process is more susceptible to tissue-induced effects: Even slight displacement or partial detachment of the ARMPatch may require realignment of the microneedle tips in the imaging field of ultrasound. A consistent imaging angle during moving actions is important for accurate length calibration, but remains challenging. In addition, the current length-calibration process relies on pixel-level segmentation and operator-dependent measurement, which remains constrained by the intrinsic resolution limits of the ultrasound probe. These factors necessitate brief manual adjustment during scanning and highlight opportunities for further optimization.

To address this issue, a promising direction to improve this method is the integration of ARMPatch with emerging wearable ultrasound platforms, enabling users to directly image the microneedles via a lightweight and epidermal ultrasound patch. Such a system could wirelessly transmit ultrasound data to a smartphone, in which advanced image processing algorithms would calibrate microneedle length and convert it into blood glucose values. Achieving this vision requires progress in two key aspects. First, codesigning the ultrasound transducer and the microneedle array would ensure consistent alignment of the imaging region with the microneedle tips, allowing rapid and reliable length calibration without manual probe positioning. Second, engineering the microneedle patch into an adhesive and conformal format would improve skin attachment and reduce motion-induced displacement, thereby supporting stable long-term monitoring. Encouragingly, recent advances in integrated microneedle-ultrasound systems for drug delivery (*77*), as well as adhesive and mechanically adaptive microneedle patches (*78*), suggest that both directions are technically feasible. These developments point toward a realistic pathway for future integration of the ARMPatch with wearable ultrasound devices.

Another emerging direction for improving the robustness of ultrasound-based readout is to advance the image-processing and calibration algorithms to reduce the errors induced by manual operation during ultrasound imaging and to improve the resolution of microneedle length calibration. In the current implementation, microneedle length is calibrated using a Sobel edge-detection method, combined with temporal averaging to reduce frame-to-frame variability. While this approach provides reliable measurements at the proof-of-concept stage, the achievable accuracy remains fundamentally constrained by the spatial resolution (15 μm per pixel) of the ultrasound probe and influenced by procedural variability, making subresolution calibration challenging. Future improvements may leverage artificial intelligence–assisted image analysis to realize the ultrasound readout stably and automatically. For example, the embedded silica microspheres can serve as point scatterers for point spread function–based super-resolution estimation, enabling subpixel and automatic localization of microneedle tips for calibrating the microneedle length. In addition, machine-learning models, such as convolutional neural networks for feature-enhanced edge detection, U-Net–style architectures for tip segmentation, and deep regression models trained to map raw pixel patterns to calibrated lengths, could further improve the accuracy by exploiting spatial information beyond predefined filters. These algorithmic advancements, together with hardware integration, are expected to substantially enhance the precision and practicality of ultrasound-readable CGM systems.

## MATERIALS AND METHODS

### Materials

Dimethyl sulfoxide (DMSO), phosphate-buffered saline (PBS), acrylamide (AAm), *N*,*N*′methylenebis(acrylamide) (MBA), 3-(acrylamido) phenylboronic acid (3-APBA), 2,2-diethoxyacetophenone (DEAP), d-(+) glucose, sodium l-lactate, sodium chloride, fructose, and galactose were purchased from Aladdin Co., Ltd. (Shanghai, China) and used without further purification. Silica microspheres were purchased from Yiyuan Biotechnology Co., Ltd. (Shanghai, China). Fluorescent silica microspheres were purchased from Beisile Co., Ltd. (Tianjin, China). The flexible resin was purchased from Jiuyue Optoelectronics Co., Ltd. (Guangdong, China). All glucose solutions used in this study refer to PBS solutions containing glucose with different concentrations to simulate physiological pH conditions.

### Preparation of hydrogel monomer solution

AAm (77.5 mol %), MBA (1.5 mol %), 3-APBA (19.5 mol %), and DEAP (1.5 mol %) were dissolved in DMSO (60%, m/v) and stirred for 12 hours at room temperature in the absence of light to prepare the glucose-responsive hydrogel monomer solution. Silica microspheres were dissolved in the hydrogel monomer solution with a ratio of 5% (v/v). The acoustically enhanced hydrogel monomer solution was stored at 4° to 8°C in the absence of light and shaken for 3 min before use. The pristine glucose-responsive hydrogel monomer was prepared in the same way without the addition of the silica microspheres.

### Hydrogel characterization

The acoustically enhanced hydrogel samples for material characterization were prepared in 2 cm–by–2 cm–by–2 cm cubic shape by curing the monomer solution in a Teflon mold under 365-nm UV light. The samples without silica microspheres were cured in the same way using the pristine glucose-responsive hydrogel monomer. Each sample was washed in PBS for more than 7 days before use (the solution was renewed every 12 hours).

FTIR results were obtained by Shimadzu IRAffinity-1S (Japan). XPS results were obtained by Thermo Fisher Scientific ESCALAB Xi+ (USA). Cryo-SEM images were obtained by Hitachi Regulus 8100 (Japan). All above characterizations were conducted by Shiyanjia Lab (Hangzhou, China).

The swelling degree of the cubic samples was measured by mass change. The samples were taken out from the PBS solution and wiped away all moisture on the surface with a dust-free cloth. After cleaning, they were weighed on an analytical balance three times. The average value of the mass was considered as the initial mass before swelling. Subsequently, the samples were immersed in glucose solutions of different concentrations, respectively. The mass of each sample after swelling from different glucose concentrations was recorded in the same way at the respective time points. At last, the swelling degree was calculated by dividing the variation in mass by the initial mass. The swelling degree of pristine glucose-responsive hydrogel was determined in the same way by measuring the mass of the hydrogel samples without silica microspheres.

### Fabrication of ARMPatch

The copper positive microneedle mold and silicone gel were purchased from Micro-nano Benteng Biotechnology Co., Ltd. (Henan, China). Liquid silicone gel was poured into the positive mold to barely cover it, and the positive mold and the silicone gel were then baked in an oven at 60°C for 1 hour. At last, a well-cured silicone gel negative mold was demolded from the positive mold.

The prepared hydrogel monomer solution was poured into the negative microneedle mold. After that, the mold was vacuumed three times (5 min per time) to ensure that the solution filled into the tips of the mold entirely. The extra monomer solution was removed from the mold carefully by pipette. Subsequently, the hydrogel microneedle part was cured under 365-nm UV light for 3 min. The flexible resin was then poured into the mold to cover the edge. The mold was vacuumed one time and placed under 365-nm UV light (1 min) for substrate curing. At last, a series of identical ARMPatches were prepared by careful demolding. The ARMPatches were immersed in PBS solutions for more than 7 days (the solution was renewed every 12 hours) to remove unreacted monomers and resin and replace DMSO with water through solvent exchange. After washing, ARMPatches were stored at room temperature, drying for further use. The microneedles without silica microspheres were obtained in the same way through the pristine hydrogel monomer. Minor fabrication variations (~20 μm) in microneedle length were observed among devices, but the calibration of SR depended on relative changes in length according to the baselines of each microneedle.

### Microneedle characterization

SEM images of microneedles were captured by TESCAN Mira 3 (USA). Optical images were captured by a camera. The height of microneedles was measured by KEYENCE VK-X200 (Japan). The distribution of fluorescent silica microspheres along a microneedle was characterized by Nikon Ti2E (Japan). The in vitro insertion test of the ARMPatch was conducted on fresh porcine tissue (purchased from a local supermarket) by keeping the ARMPatch inserted into porcine tissue for 10 s and capturing the tips and the array before and after insertion via the optical microscope (Nikon Ti2E) and a high-resolution camera.

The compression test was conducted by Instron E10000, and the value of displacement and the force were read via a standard program. An ARMPatch was placed flat on the underlying base with the microneedle tips facing upward. We first adjusted the position of the platen and kept it merely touching the tips of the microneedles. Subsequently, we initialized the force and displacement to zero and started the program. The platen was displaced downward to press the microneedles at a speed of 10 mm/min. The compression procedure was finished in 9 s, and the curve of displacement-force was obtained. We observed the microneedles again to ensure no fractures appeared on them. Microneedles with and without silica microspheres were tested and analyzed.

### In vitro imaging and test

The in vitro glucose-sensing experiments of the ARMPatch were tested in glucose solutions and agarose gel phantoms. Glucose solutions of different concentrations were prepared in PBS, and potential interfering substances were added to the corresponding groups. The solution was poured into a small petri dish, and an ARMPatch was then placed in the dish with the microneedle tips facing up. The height of the solution is further above the microneedle tip. A standard ultrasound imaging equipment (FUJIFILM Vevo 2100) was used to image the device. A probe (40 MHz) was immersed in the solution, and its position was adjusted so that the microneedle was clearly visible in the ultrasound image. After the patch was placed, the probe was quickly set up and the cross-sectional image of one array of the microneedles at this moment was captured to calibrate the initial lengths of the microneedles. Following that, the images of the microneedles after the interaction with glucose were captured based on different conditions (time, concentration, and selectivity). The lengths of the microneedles were measured using ImageJ software according to the resolution of the ultrasound images provided by the equipment. The SRs of ARMPatches under different conditions were determined by dividing the length variation by the initial length. Three microneedles of an array were randomly selected to calculate the average value of SR for one device.

The tests in agarose gel phantom were proposed to simulate imaging and constraints in tissue. The initial length of microneedles was captured under the microscope before phantom curing. Following that, the agarose gel was prepared by first dissolving agarose powder in a boiled PBS solution (1%) with a respective quantity of glucose. Subsequently, the mixture, before completely cooled, was poured into a glass dish where an ARMPatch had already been placed. In this way, the agarose gel phantom can cover the entire ARMPatch for glucose-sensing. Subsequently, the ultrasound probe was positioned on the surface of the phantom to scan and image the microneedles in the phantom. Thus, the final state of corresponding microneedles was captured via ultrasound after 1 hour and the SR could be calculated in the same way (division) mentioned in the above.

To simulate continuous monitoring of diabetic patients’ blood glucose level, glucose solutions with concentrations of 4, 12, 6, and 20 mM were prepared to mimic glucose levels under fasting, postprandial, postabsorptive, and abnormal postprandial conditions, respectively. The lengths of the ARMPatch were captured via the ultrasound probe at the proposed time point respectively as shown in the result. The SRs were calculated in the same way as mentioned above.

The durable stability of the ARMPatch was evaluated by immersing a device in a 12 mM glucose solution (mimicking a postprandial condition) for 1 hour each day and repeating the measurement every day for a continuous period of 56 days. The initial state of the microneedles was determined on the first day, and the final states within 56 days were determined every day after 1 hour of immersion. Every day after the SR for 12 mM glucose was recorded, the ARMPatch was kept in PBS for recovery. Last, the ARMPatch was placed in 14 and 10 mM glucose solutions to verify its consistent glucose-sensing ability after 56 days. The SRs were calculated in the same way as mentioned above.

### Biocompatibility test

The cytotoxicity of the synthesized hydrogels was evaluated by the cell counting kit-8 (CCK-8) test. After sterilization, the hydrogel samples were treated with cell culture medium (0.1 g/ml) and shaken at 37°C for 24 hours, and the extract was then gathered after filtration. The cell medium without any treatment was used as the blank control group. Consequently, L929 cells were seeded in 96-flat plates (Greiner). After incubation for 24 hours, the cell culture medium was replaced with 200 μl of extract medium or control medium and incubated for 24 and 48 hours. At each time point, the cell viability was measured using a CCK-8 assay kit (Servicebio, China) according to the manufacturer’s instructions for five times, and the results were recorded by the operator.

Histopathological staining experiments were used to evaluate the immune response induced by microneedles. An ARMPatch with sterilization was fixed on the mouse skin for 8 hours and removed. The immune response induced by the ARMPatch was obtained on the first day, and the spontaneous recovery results were obtained on the fourth and seventh days. The targeted tissue sections were removed, fixed with paraformaldehyde, and embedded in paraffin to prepare sections, and then stained with CD3 for T cells and F4/80 for macrophages. Subsequently, the tissue sections were evaluated by a microscope. The F4/80-positive macrophages and CD3-positive T cells were observed at a field of view of 400 times, and three different fields were randomly selected for statistics. Immunoreactive cells were quantified as the mean percentage of positive cell area. Both cytotoxicity and histopathology experiments were conducted and counted by Huateng Biotech Co., Ltd. (Guangzhou, China).

The chemical stability of the hydrogel was evaluated by immersing a hydrogel sample in deionized water. Specifically, a cubic hydrogel sample was soaked in deionized water for 7 days, after which the sample was removed and the elemental composition of the solution was analyzed. A separate deionized water sample was also examined as a standard control. ICP-MS was used to detect silicon and boron, while TOC analysis was used to measure organic carbon and nitrogen. Both tests were conducted by Shiyanjia Lab (Hangzhou, China).

### In vivo imaging and test

The in vivo blood glucose level monitoring via ARMPatch was performed using 12 male nude mice (4 to 8 weeks). All experimental procedures on live animals (mice) were carried out in line with the approved protocols of Shenzhen Institute of Advanced Technology (SIAT-IACUC-250307-YGS-ML-A2924). Experimental design was optimized to reduce the number of animals needed, and all efforts were made to minimize animal suffering and distress throughout the experimental procedures. A series of identical ARMPatches after sterilizing were cut into a 5-by-5 array for applying. All mice were fasted for 6 hours before the experiment. The mice were anesthetized with isoflurane (1 to 2%) during ultrasound imaging and placed on a 38°C heating plate to maintain body temperature.

For ultrasound imaging, an ARMPatch was inserted into the abdomen of the mouse from the side, with the tips of the microneedles facing up. The ultrasound probe was moved downward until it was in contact with another side of the mouse, and the probe was gently adjusted to fix the probe, the mouse body, and the ARMPatch by slight pressing. The ultrasound probe was then fixed at the height and slowly moved horizontally until the microneedles were observable in the ultrasound image, and then the probe was completely fixed to maintain the image of the microneedles. The images of the microneedles were then captured as the initial length data. The initial lengths of the microneedles applied to six mice were captured in this way, respectively. Following that, 50% glucose solution was injected into the mice by intraperitoneal injection. The solutions with doses of 0, 5, 6, 7.5, 9, and 10 ml/kg were injected, respectively, to induce different blood glucose levels for six mice. After 60 min, a commercial glucose meter (ACCU-CHEK) was used to monitor the blood glucose by the tail vein, representing the standard glucose levels of different mice. Last, ultrasound images of the ARMPatches were captured at this state as the final state. The images were analyzed in ImageJ software, and one observable microneedle was selected to calculate SR in the same way in terms of the length variation and initial length. The length was obtained by the average length in three ultrasound images obtained in a short time interval of the same microneedle.

The continuous blood glucose test was performed in a similar way. The ARMPatch, ultrasound probe, and the mouse were fixed in the same way. The glucose was injected into the mice with 5 or 10 ml/kg for the two groups. After the first 1-hour period, the standard glucose level was measured, and ultrasound images were captured. Subsequently, insulin (in 0.9% NaCl solution) was injected into the mice with 0.1 U/kg. After one more hour of blood glucose declining, the standard glucose level was measured again, and the ultrasound images were captured again in the same way to analyze the SRs of the second declining stage.

The 7-day continuous experiment was primarily conducted by protecting the ARMPatch from detaching using medical adhesive tape. The mouse remained active and awake throughout the 7 days. During ultrasound imaging, the tape was removed, and the mouse was anesthetized. On day 5, an insulin solution was administered to the mouse with a dose of 0.1 U/kg. On day 7, a glucose solution was administered to the mouse with a dose of 10 ml/kg. Other ultrasound imaging and standard blood glucose measurement procedures were performed as previously described. On day 7, the ARMPatch was removed, and the skin recovery and overall health status of the mouse were examined on the following day.

The in vivo changes in the SR of the ARMPatches obtained via the microscope were operated in the same way. Briefly, the microneedles of three identical ARMPatches were captured via a microscope before insertion to record the initial length. Following that, the ARMPatches were attached to three mice, respectively, and kept for 1 hour. The different blood glucose levels in the mice were induced in the same way mentioned above. After that, the patches were removed, and the blood glucose levels were measured with the commercial glucose meter for comparison. Last, the images of the corresponding microneedles were captured again under an optical microscope in a short time to calibrate the length and determine the SR.

### Calibration of length

The lengths of the microneedles in the ultrasound and microscope images were determined by the blinded measurement of the authors via ImageJ software. For the situation of microneedles in glucose solution, the images were first treated with the “Find Edges” process in ImageJ to distinguish the microneedle pattern from the background and then calculate the length between tips and substrate. For the measurement of the microneedles in animal models and in skin phantoms, the lengths of the microneedles were calibrated directly from the images without preprocessing. For in vitro tests, the lengths of three microneedles in a microneedle array simultaneously captured via ultrasound were selected to ensure reliability. For in vivo tests, the three values of the microneedle length in animal experiments were calibrated via three different ultrasound images of one microneedle captured in a short time interval. There may be errors in the calibration standards of different situations according to the complicated imaging conditions, but it is essential that the length calibration standards for the recognition of the same microneedle before and after the response remain consistent.

## Supplementary Materials

### The PDF file includes:

Sections S1 to S3 Figs. S1 to S18 Tables S1 to S3 Legends for movies S1 to S3 References
