# Supplementary material for: Augmenting ultrasound for continuous glucose monitoring via a wearable acoustically readable microneedle patch
Source: Sci Adv. 2026 May 1;12(18):eaec3209. doi: 10.1126/sciadv.aec3209 (PMC13134621; doi:10.1126/sciadv.aec3209)
Supplement: Supplementary file 1 — Sections S1 to S3 Figs. S1 to S18 Tables S1 to S3 Legends for movies S1 to S3 References [file sciadv.aec3209_sm.pdf]

Science Advances
AAAS

Supplementary Materials for

Augmenting ultrasound for continuous glucose monitoring via a wearable acoustically readable microneedle patch

Wanglinhan Zhang *et al.*

Corresponding author: Jae-Woong Jeong, jjeong1@kaist.ac.kr; Long Meng, long.meng@siat.ac.cn; Zhongqing Su, zhongqing.su@polyu.edu.hk

Sci. Adv. **12**, eaec3209 (2026)
DOI: 10.1126/sciadv.aec3209

The PDF file includes:

Sections S1 to S3
Figs. S1 to S18
Tables S1 to S3
Legends for movies S1 to S3
References

Other Supplementary Material for this manuscript includes the following:

Movies S1 to S3

Section S1

Optimization of the Response Time of the ARMPatch for CGM

Although the current 60-minute response time of the ARMPatch is sufficient to provide clinically meaningful glucose-trend tracking for users, further reduction of this response time may facilitate more precise monitoring and earlier diagnosis of dangerous glycemic events. Therefore, optimizing the response time of acoustically readable hydrogel microneedles remains valuable for future clinical translation. Based on our present findings, three potential optimization directions can be implemented.

Firstly, optimization of the glucose-responsive hydrogel composition would accelerate solvent diffusion. We investigate the effect of the proportion of embedded silica microspheres in the hydrogel matrix by fabricating microneedles with the same geometry but containing different concentrations of silica microspheres and recording the SRs of these microneedles in response to a 10 mM glucose solution over time. Defining the time required to reach 90% of the maximum swelling ratio as the response time, microneedles containing 2%, 5% and 10% silica microspheres exhibit response time of 50, 60 and 120 minutes to the glucose concentration of 10 mM, respectively (**Fig. S15A**). The results indicate that reducing the concentration of silica microspheres in the hydrogel allows hydrogel microneedles to reach a quasi-saturated swelling more rapidly, effectively shortening the response time. This can be attributed to the function of silica microspheres as an additional crosslinker in the hydrogel; lowering their content decreases the overall crosslinking density, thereby accelerating solvent diffusion and enhancing glucose responsiveness. However, the decrease in the concentration of silica microspheres may in the meantime diminish the acoustic contrast of the microneedles in ultrasound images, potentially compromising readability. Future optimization requires adjusting the concentration of silica microspheres embedded in the hydrogel according to the mechanical density of the target tissue, balancing acoustic contrast with response-time performance.

Secondly, refining the microneedle dimensions is also considered to shorten the response time. Reducing volume is a well-established strategy for shortening the swelling-equilibrium response time of hydrogels. Based on our chemical–mechanical coupling model for hydrogel microneedle swelling, we simulate the swelling of microneedles of different volumes under identical chemical parameters in response to a 10 mM glucose. As microneedles of different volumes are designed

with a fixed aspect ratio between their length and base dimensions, changes in volume result in proportional changes in length, making the length as a valid parameter for calculating SRs. To reach a quasi-saturated swelling (90%), it takes 27, 40 and 52 minutes for the microneedles with the initial length of 800, 1000 and 1200 μm, respectively (**Fig. S15B**). The results show that smaller microneedles reach a quasi-saturated swelling more rapidly and thus require less time to respond to changes in glucose concentration. However, decreasing microneedle size inevitably reduces the absolute magnitude of length change, which may challenge the resolution limits of ultrasound imaging. Consequently, the microneedle size used in clinical applications should be optimized in conjunction with the resolution of the ultrasound system to ensure reliable readout while achieving faster response time.

Beyond material and geometric optimization, algorithmic prediction approaches offer a complementary strategy to address the intrinsic delay associated with hydrogel swelling. The swelling kinetics of glucose-responsive hydrogels typically follow a monotonic, diffusion-limited trajectory that can be well described using the first-order or stretched-exponential kinetic models. As a result, the early-time SR evolution (e.g., the first 10–20 minutes) contains sufficient information to estimate the kinetic parameters governing the swelling process. By fitting these initial SR data to parametric models, the equilibrium SR can be forecasted prior to the hydrogel reaching its quasi-saturated swelling state. In addition to classical kinetic fitting, the data-driven regression methods (e.g., nonlinear least-squares regression, autoregressive forecasting, Gaussian-process regression or lightweight machine-learning regressors) may further enhance prediction accuracy by capturing subtle variations in swelling dynamics across different hydrogel formulations or physiological environments. Once the predicted saturated-state SR is obtained, it can be directly mapped to the corresponding glucose concentration using the established calibration curve, thereby enabling an earlier estimate of blood glucose level even when the hydrogel is undergoing initial swelling. Such algorithmic prediction frameworks can substantially shorten the effective response time of the ARMPatch, offering a promising direction for future clinical translation in rapid glucose-trend estimation.

Section S2

Chemical–mechanical coupling model for the swelling of hydrogel microneedles

The swelling of the phenylboronic acid (PBA) hydrogel in the presence of glucose can be described by a Multiphysics model derived via the non-equilibrium thermodynamic theory. The model consists of a set of equations (Eqs. (1) – (10)) that account for force balance, chemical equilibrium, and swelling degree in the hydrogel (73).

In particular, the force balance can be described as

$$-\boldsymbol{\nabla} \cdot \boldsymbol{\sigma} = 0 \tag{1}$$

$$\boldsymbol{\sigma} = \frac{NkT}{\det\boldsymbol{F}}(\boldsymbol{F}\boldsymbol{F}^{T} - \boldsymbol{I}) - \Pi\boldsymbol{I} \tag{2}$$

where $\boldsymbol{\sigma}$ is the total stress tensor; $\boldsymbol{F}$ is the deformation gradient of the polymer network; $\boldsymbol{F}^{\boldsymbol{T}}$ signifies the transpose of the deformation gradient; Π is the osmotic pressure in hydrogel; N is the number of polymer chains per unit polymer volume, k is the Boltzmann constant, T is the temperature, and $\boldsymbol{I}$ is the second-rank identity tensor.

The chemical equilibrium of the hydrogel is governed by the chemical potential of the solvent molecules in the hydrogel, described as

$$\mu = kT\left(\ln cv^{s} + (1 - cv^{s}) + \chi(1 - cv^{s})^{2} - \frac{fNv^{s}(1-cv^{s})}{cv^{s}}\right) + \Pi v^{s} \tag{3}$$

where c is the solvent molecule concentration in the current configuration, χ denotes the polymer-water interaction parameter, f is the number of charges per polymer chain in the network, and v^{s} is the volume of a solvent molecule with s standing for the solvent.

The swelling degree of the hydrogel is constrained by the incompressibility of both the polymer network and the solvent molecules, defined as

$$1 + cv^{s}J = J \tag{4}$$

where $J = \det\boldsymbol{F}$ is the determinant of the deformation gradient of the polymer network.

The swelling kinetics of the hydrogel, i.e., hydrogel swelling as a function of time, can be described by the solvent transport equations, defined as

$$\frac{\partial c}{\partial t} + \boldsymbol{\nabla} \cdot \boldsymbol{j} = 0 \tag{5}$$

$$\boldsymbol{j} = -\frac{Dc}{kT}\nabla\mu \tag{6}$$

where $\boldsymbol{j}$ denotes the solvent flux and D is the diffusivity of the solvent molecules. In the simulation, D is taken to be a function of the determinant of the deformation gradient of the polymer network similar to one previous report (55), defined as

$$D = \frac{D_{\mathrm{h}} - D_{\mathrm{l}}}{1+\exp\left(-\frac{2(J-J_{\mathrm{mid}})}{J_{\mathrm{range}}}\right)} + D_l \tag{7}$$

where D_{h}, D_{l}, J_{mid}, and J_{range} are fitting parameters.

To account for the glucose sensing mechanism, we start with the disassociation equilibrium of the boronic acid functional group, *i.e.,* -$B(OH)_2$, in the PBA-based hydrogel, defined as

$$-\mathrm{B(OH)_2} + \mathrm{H_2O} \rightleftharpoons \mathrm{H^+} + -\mathrm{B(OH)_3^-} \tag{8}$$

where the equilibrium concentration of the chemical species $-\mathrm{B(OH)_2}$, $\mathrm{H^+}$ and $-\mathrm{B(OH)_3^-}$ is dictated by the pKa value defined as $\mathrm{p}K\mathrm{a} = -\log\frac{[\mathrm{H^+}][-\mathrm{B(OH)_3^-}]}{[-\mathrm{B(OH)_2}]}$. The $[\cdot]$ means the mole concentration of the species. Importantly, the $-\mathrm{B(OH)_3^-}$ species are bound on polymer chains, endowing the polymer network a net charge density. In the presence of glucose, the $-\mathrm{B(OH)_3^-}$ species bind selectively to glucose, according to the following equation, to form a charged complex (illustrated in **Fig. 1B**):

$$\mathrm{Glucose} + -\mathrm{B(OH)_3^-} \rightleftharpoons \mathrm{Complex^-} \tag{9}$$

The glucose binding equilibrium is characterized by its binding constant defined as $K_a = \frac{[\mathrm{Complex^-}]}{[\mathrm{Glucose}][-\mathrm{B(OH)_3^-}]}$. The binding of glucose shifts the equilibrium of Eq. (8) to right, as a result of which more -$B(OH)_2$ functional groups disassociate, and therefore the overall charge density (*i.e.*, $[-\mathrm{B(OH)_3^-}] + [\mathrm{Complex^-}]$) increases as the glucose concentration increases. This chemical reaction changes the parameter f in Eq. (3) in accordance with

$$f = f_{\mathrm{tot}}\frac{1+K_a\times[\mathrm{Glucose}]\times10^{-3}}{1+K_a\times[\mathrm{Glucose}]\times10^{-3}+10^{(\mathrm{p}K_a-\mathrm{pH})}} \tag{10}$$

where f_{tot} is the total number of -$B(OH)_2$ functional groups per polymer chain. [Glucose] is the glucose centration in the unit of mM. Eq. (10) underscores that different glucose concentrations

will lead to different f, which results in different swelling degrees of the hydrogel through Eqs. (1) – (4) and different hydrogel swelling kinetics through Eqs. (5) and (6). It is noted that body fluid is buffered with a pH = 7.4.

To summarize, the model developed above has a set of parameters, including N, χ, f_{tot}, Ka, pKa, D_{h}, D_{l}, J_{mid}, and J_{range}, which determine the swelling degree of the hydrogel as a function of the glucose concentration and time. In this model, N and f_{tot} can be calculated by the chemical recipe used during the hydrogel synthesis. In addition, we assume that for a given hydrogel, χ, Ka, and pKa values are fixed, hence we treat them, D_{h}, D_{l}, J_{mid} and J_{range} as fitting parameters.

Eqs. (1) – (10) form a full model capable of solving both equilibrium and kinetical hydrogel swelling in large deformation. We developed a solver to solve Eqs. (1) – (10) based on MOOSE, which is an open-source C++ finite element framework (79). The geometry used in the simulation replicates the pyramid-shaped microneedle, as shown in **Figure S16**. The bottom of the microneedle is constrained in the simulation. Other surfaces are free of stress when studying microneedle swelling in glucose solution. To simulate the swelling in the constrained scenario, a hydrostatic pressure of $0.05kT/v$ is applied as a boundary condition on these surfaces to model the pressure exerted by surrounding tissue on the microneedles.

In the simulation, Nv^s and f_{tot} are fixed at 0.02 and 6.0, respectively, according to the recipe used in the hydrogel synthesis. Other parameters are determined by comparing the simulation results with the experimental ones iteratively across multiple glucose concentrations. Specifically, we first obtained experimental results on the swelling of the PBA hydrogel under the modulation of 0, 5, 10, 15, 20, 30, and 40 mM at the time point of 60-minuate. Then we tuned χ, Ka, and pKa, as well as D_{h}, D_{l}, J_{mid}, and J_{range}, iteratively, until the simulation results from the same set of parameters matched all the experimental hydrogel swelling results across the respective glucose concentrations. The fitted parameter values are determined and listed in **Table S3**. All of them are within a reasonable range (55, 80). **Figure S17** shows that the simulated f value fits well with Eq. (10). Using the same set of parameters to match all the experimental results ensures high fidelity of our model.

To further validate the consistency between our experimental observations and theoretical modeling, we compare the hydrogel swelling kinetics over time as obtained from both simulation and experiment (**Fig. S18**). By applying the boundary condition, the swelling degree of the

hydrogel under constraints can also be determined. With this model and the derived parameters, we are able to predict the swelling behavior of hydrogel microneedles under various conditions, including concentration-dependent swelling, time-dependent swelling and constraint-modulated scenarios. Detailed comparisons between simulated and experimental results are provided in the Results section, demonstrating a strong agreement and thereby validating the theoretical framework for analyzing the swelling behavior of hydrogel microneedles.

Looking ahead to broader applications, the parameters within this model are tunable to accommodate a wide range of stimulus-responsive hydrogels. For instance, different chemical equilibria can be incorporated by adjusting binding constants of respective biomarkers, and diverse material compositions can be modeled by modifying mechanical parameters. This flexibility enables the analysis of swelling behaviors in alternative microneedle devices designed for healthcare applications, as well as under different physiological constraints imposed by surrounding tissue. The predicted swelling behavior of microneedles can also serve as a reference to calibrate the information extracted from ultrasound imaging, enhancing the interpretability and quantitative accuracy of the sensing readouts. Overall, the model provides a versatile and predictive tool for optimizing this ultrasound-based microneedle biosensing technology.

Section S3

Statistical analysis of the correlation between ultrasound-calibrated SRs and blood glucose levels

In total, eight samples are included in the statistical analysis, consisting of six mice undergoing independent glucose monitoring and two mice from the rising phase of the glucose-insulin cycling experiments. Data from the latter two mice are incorporated in the statistical analysis, considering their experimental conditions of glucose-rising are completely consistent with those of the independently tested mice. Due to the difficulty in practice to maintain a strictly constant glycemic state in nude mice, a stable control group is not established. Instead, Pearson correlation analysis is employed to evaluate the correlation between ultrasound-calibrated SRs and blood glucose levels from eight samples. Despite inter-individual variability, the in vitro results indicate that the SR should follow an approximately linear relationship with glucose concentration.

Using the ultrasound-calibrated SRs and corresponding blood glucose levels from all eight samples, the Pearson correlation coefficient is calculated as $R = 0.942$, with a coefficient of determination of $R^2 = 0.886$. This reflects a strong linear correlation between the SR and blood glucose level, while the presence of a few outliers is expected and acceptable given inter-individual biological variability. A two-tailed significance test yields a t-value of 6.84, corresponding to a p-value of 0.0005. Therefore, the linear correlation between the ultrasound-derived SR and blood glucose levels is statistically significant in a group containing eight samples, supporting the in vivo reliability of this approach for blood glucose level calibration.

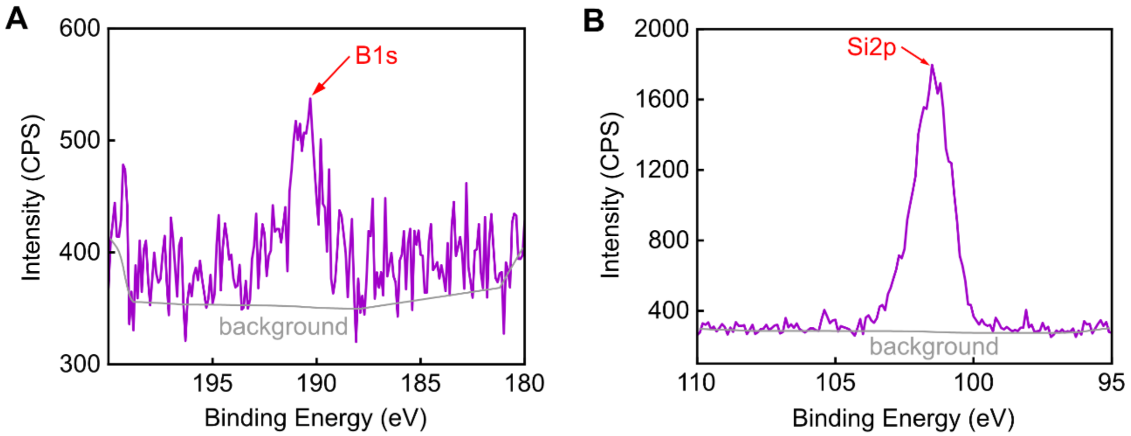

Fig. S1. Enlarged figures of the specific peaks in XPS analysis. (**A**) Enlarged figure of B1s peak in the XPS result. (**B**) Enlarged figure of Si2p peak in the XPS result.

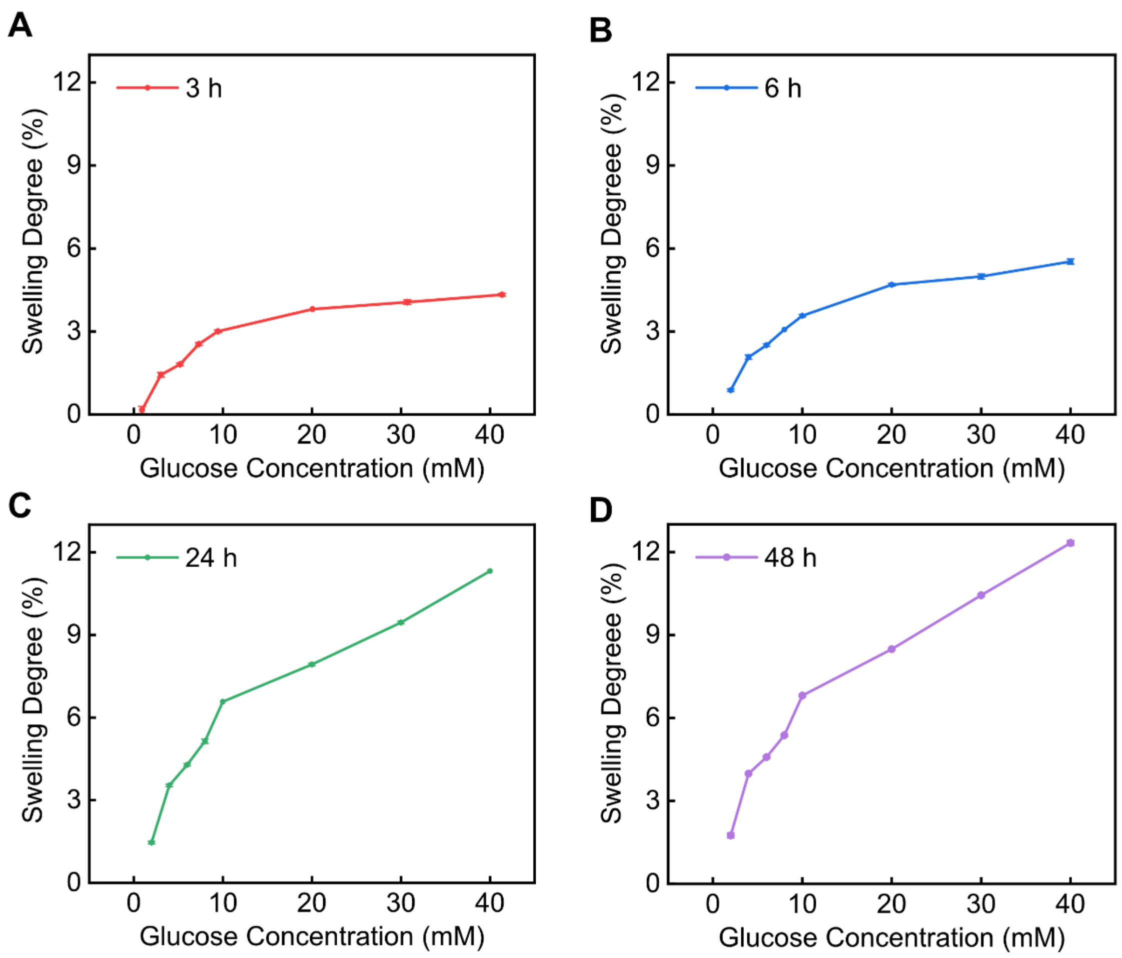

Fig. S2. Glucose-responsiveness of the fabricated cubic hydrogel samples. (**A** to **D**) Swelling degree of hydrogel samples responding to the changes in glucose concentration within 3 h (A), 6 h (B), 24 h (C) and 48 h (D). Error bars refer to standard deviation (SD), n=3.

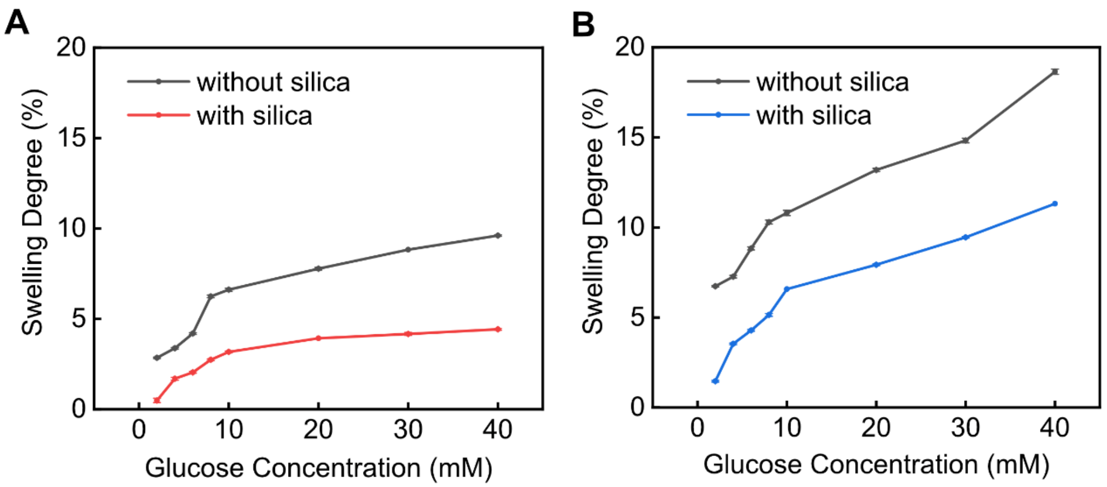

Fig. S3. Effect of silica microspheres on the glucose-responsiveness. (**A** and **B**) Comparison of the response of the hydrogel samples with and without silica microspheres in 3 h (A) and 24 h (B). Error bars refer to SD, n=3.

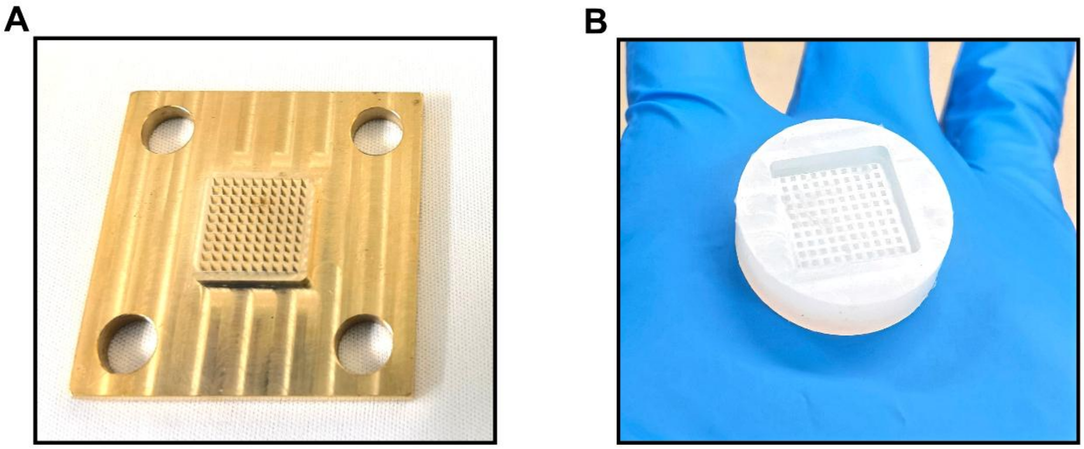

Fig. S4. Molds for ARMPatch fabrication. (**A**) Optical image of the positive mold. (**B**) Optical image of the negative mold.

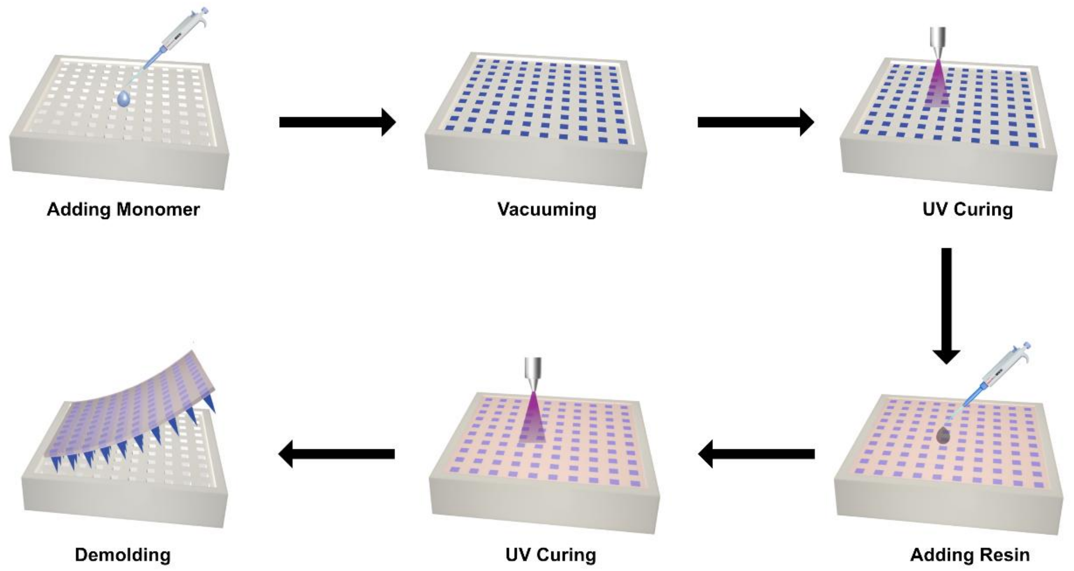

Fig. S5. Fabrication procedures of the ARMPatch of a 10×10 microneedle array.

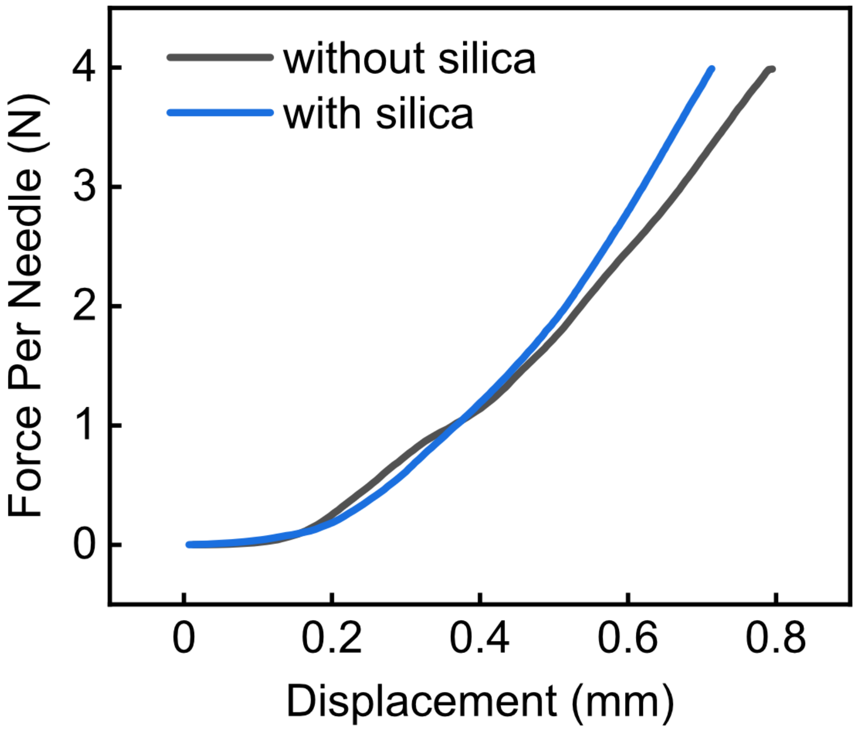

Fig. S6. Comparison of the mechanical strength of the microneedles with and without silica microspheres.

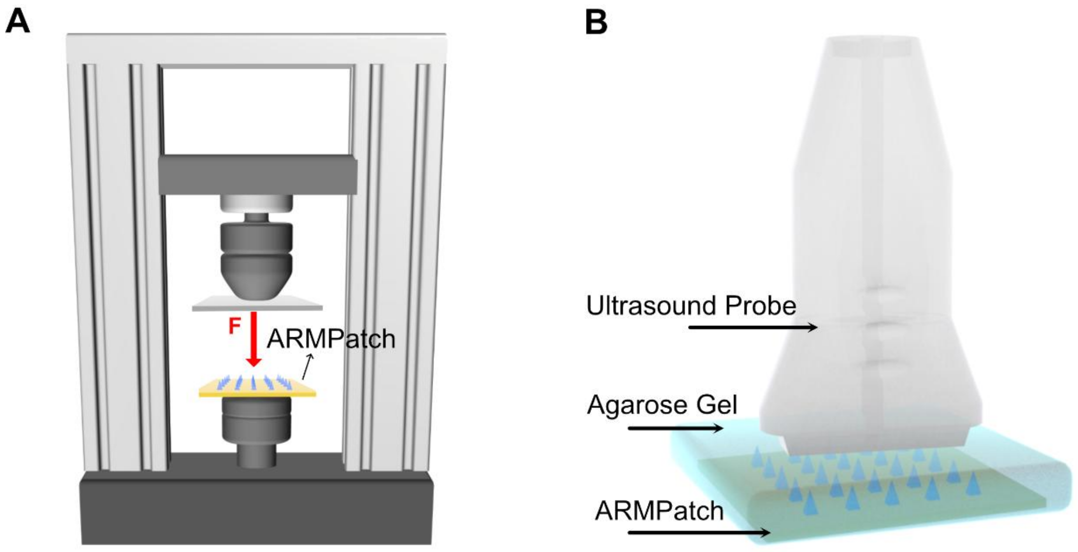

Fig. S7. Experiment setup. (**A**) Mechanical strength test for microneedles. (**B**) Ultrasound imaging on the microneedles in a skin phantom using a standard ultrasound probe.

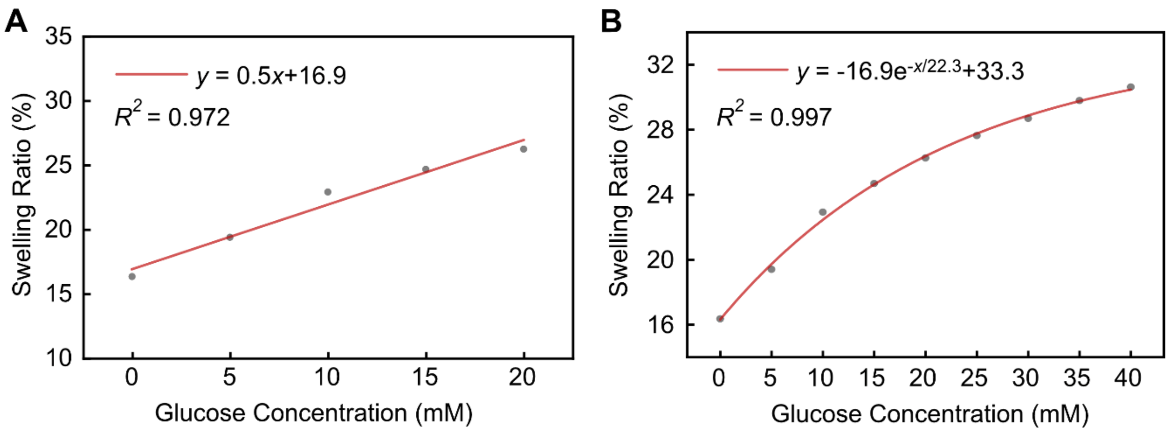

Fig. S8. Analysis of the sensitivity and detection limit. (**A**) Linear fitting of the SR and glucose concentrations in the range of 0-20 mM (general physiological range) to determine the detection limit. (**B**) Non-linear fitting of the SR and glucose concentrations in the range of 0-40 mM.

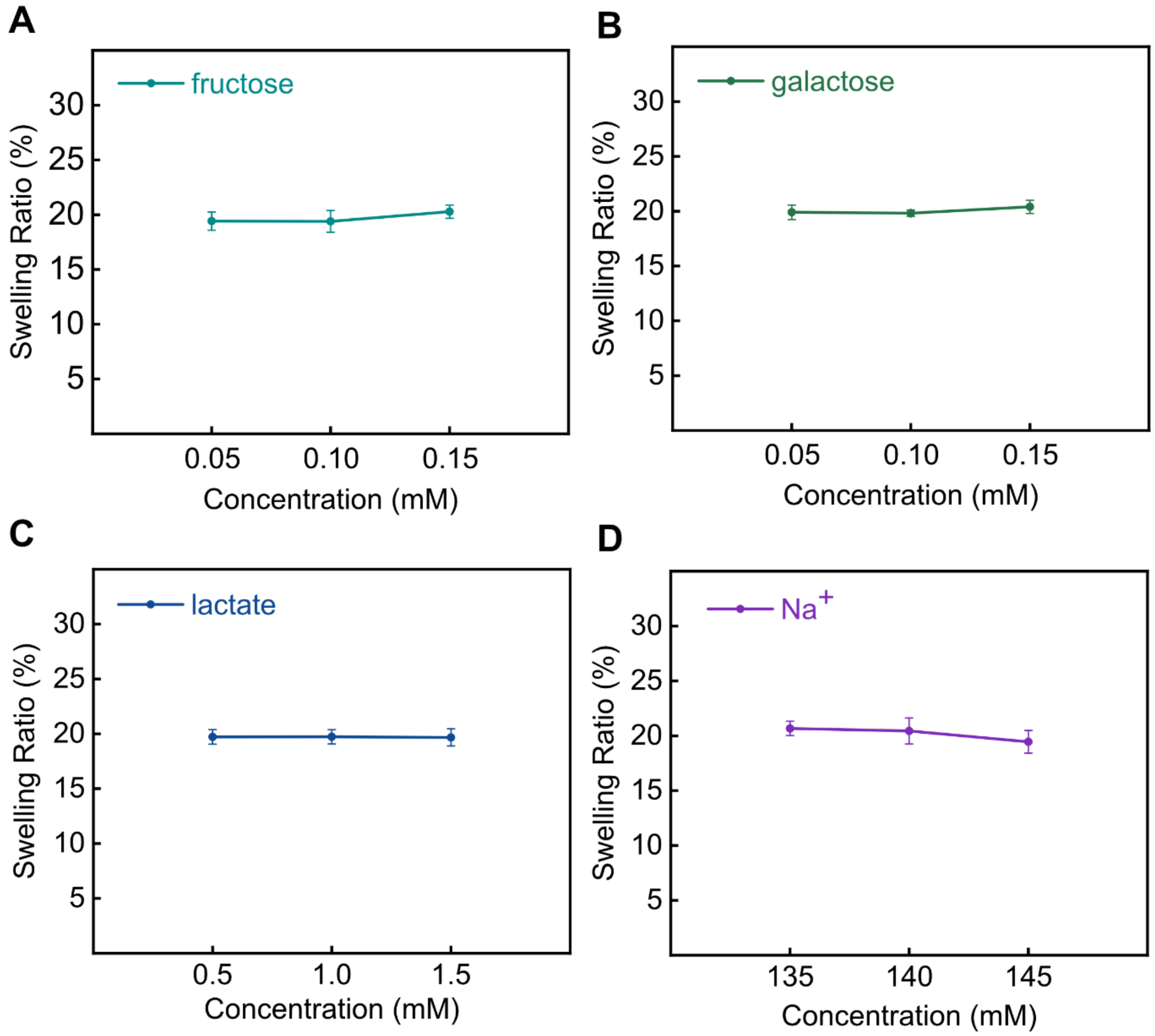

Fig. S9. Bioselectivity of the ARMPatch. (**A** to **D**) Correlations between the SR and the concentration of interferences, including fructose (A), galactose (B), lactate (C) and Na^+ (D) in the bioselectivity test. Error bars refer to SD, n=3.

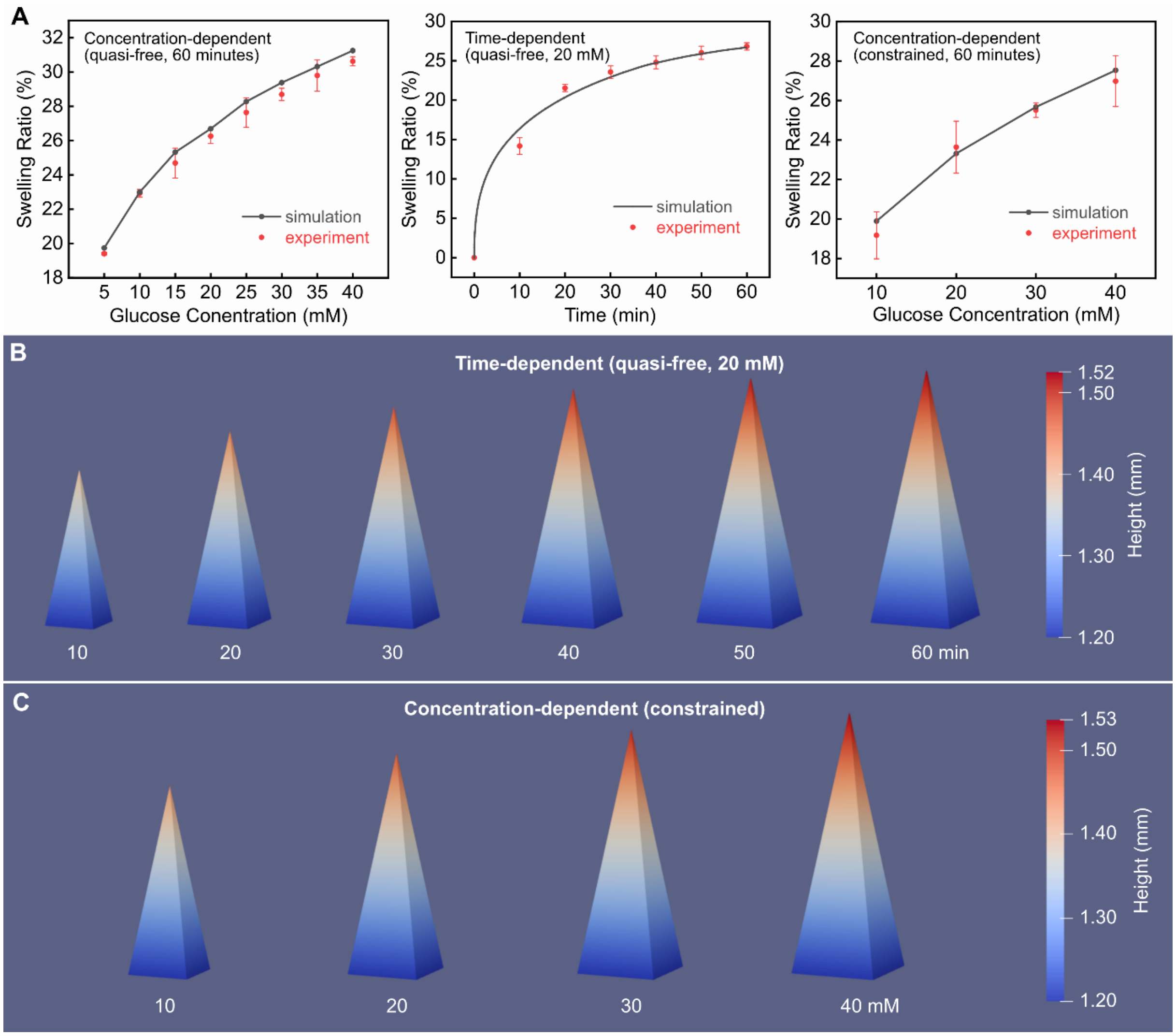

Fig. S10. Simulation results of the SR against different conditions. (**A**) Comparison of the simulated and experimental SRs varying with glucose concentrations and time in the quasi-free and the constrained scenario. (**B**) Simulation results of the time-dependent swelling of the microneedles against 20 mM glucose. (**C**) Simulation results of the concentration-dependent swelling of microneedles in the constrained scenario. Error bars refer to SD, n=3.

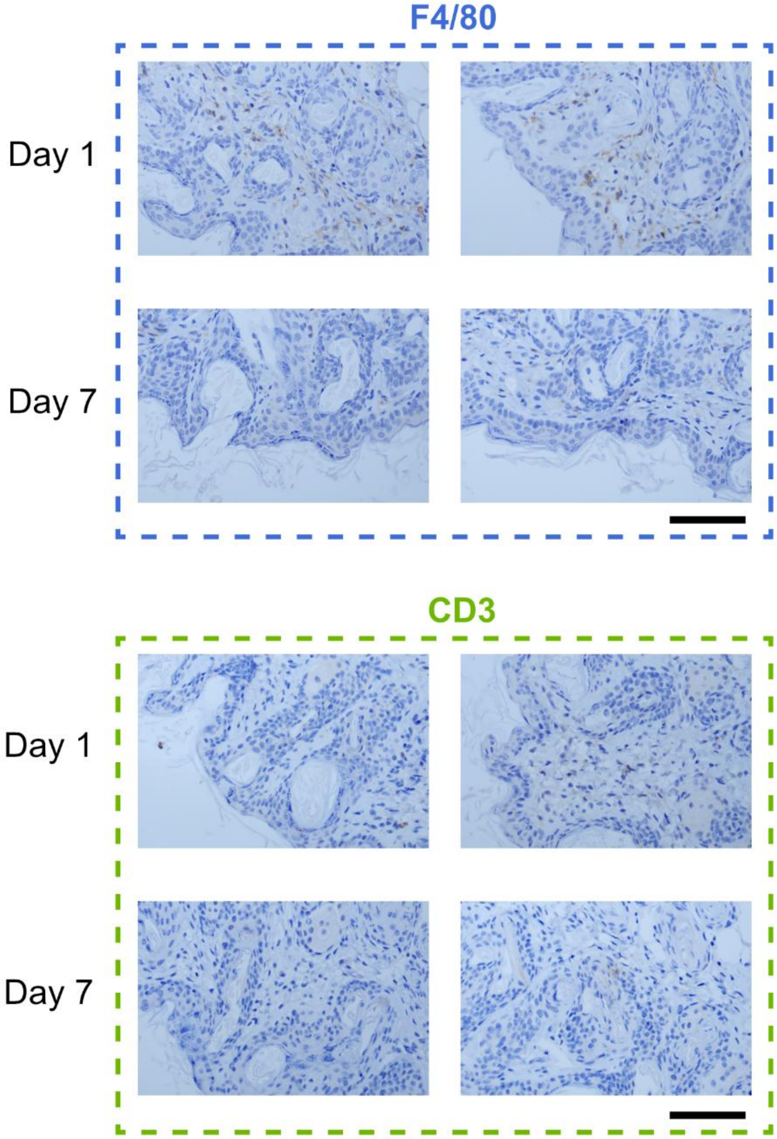

Fig. S11. Supplementary results of the immunostaining of mouse tissue sections induced by the ARMPatch. Scale bar, 50 μm.

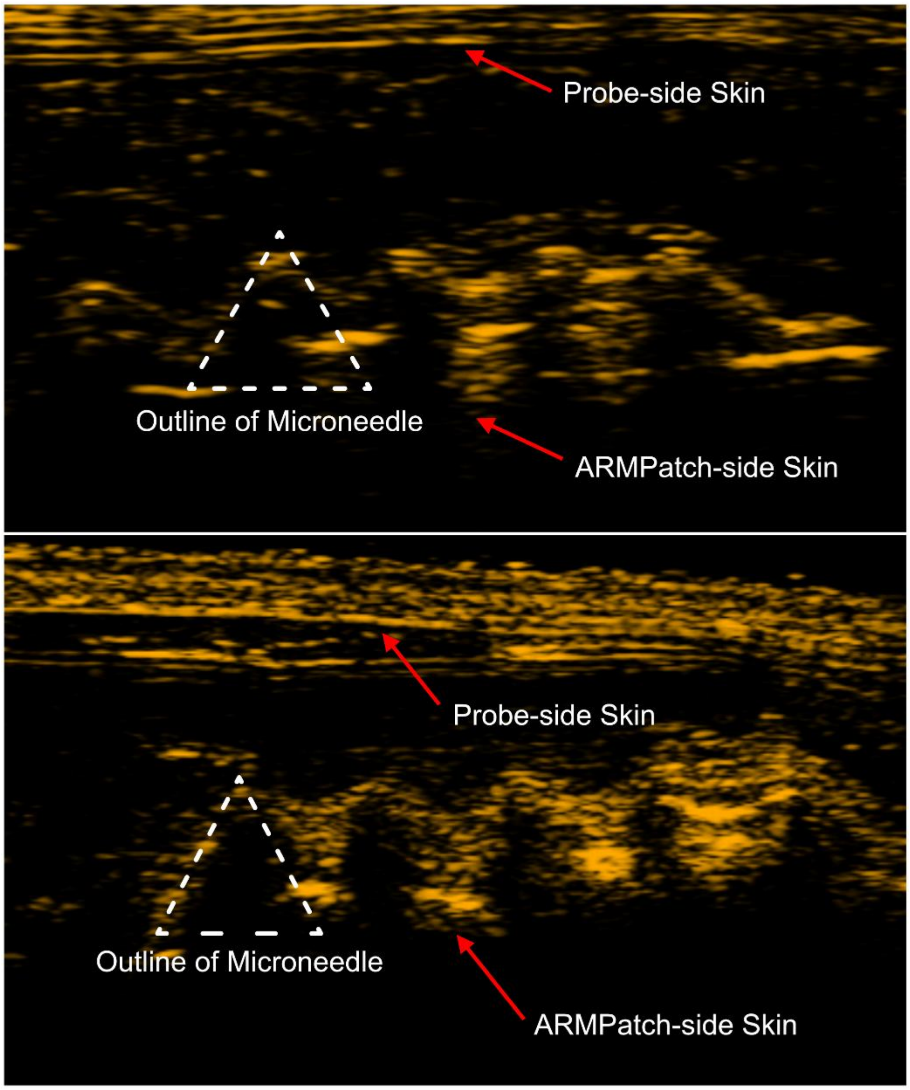

Fig. S12. Supplementary ultrasound images of the microneedles of the ARMPatch penetrating the epidermis in vivo. Pseudo-color is achieved by modifying LUT using the image editing software.

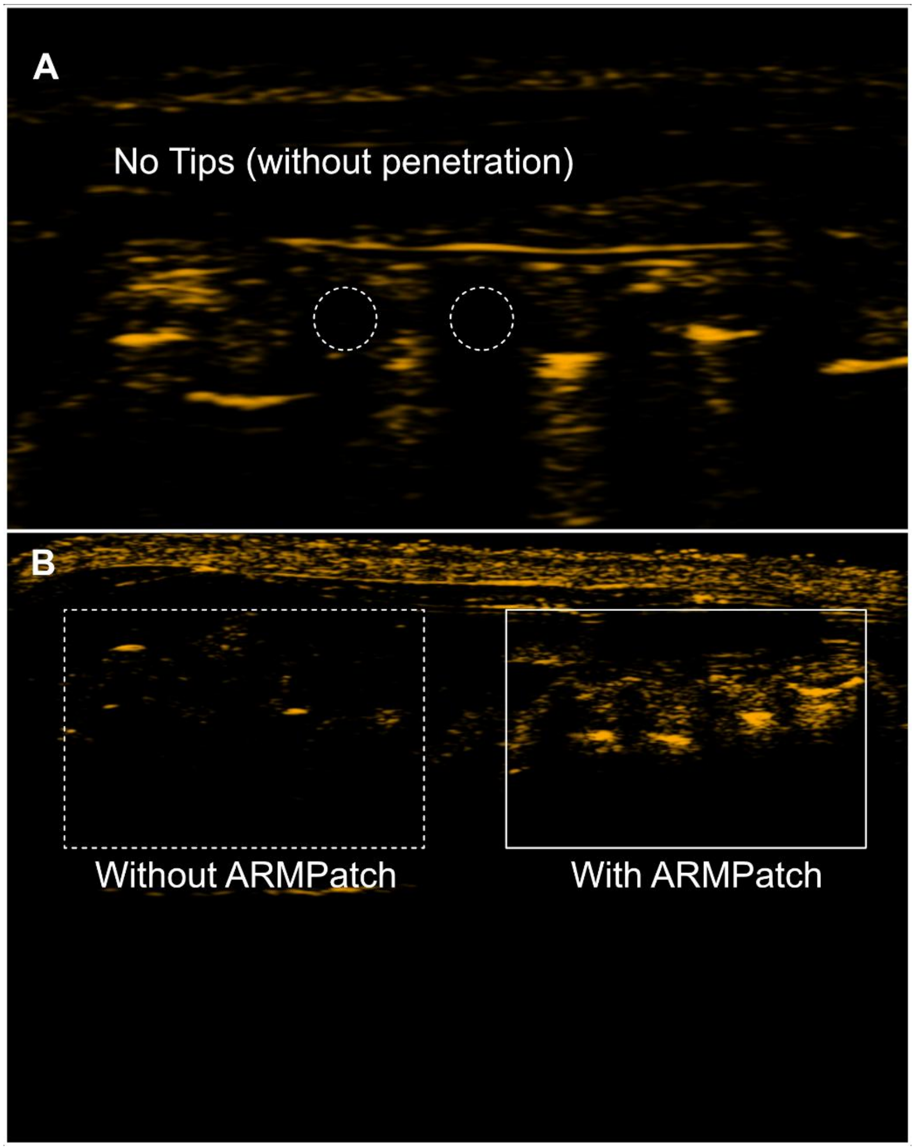

Fig. S13. Controlled experiment of ultrasound imaging. (**A**) Ultrasound image of the tissue when the ARMPatch only applies to the mouse with sufficient contact instead of penetrating. (**B**) Comparison of the ultrasound image of the internal cavity of the mouse with and without the ARMPatch simultaneously in a wide view. Pseudo-color is achieved by modifying LUT using the image editing software.

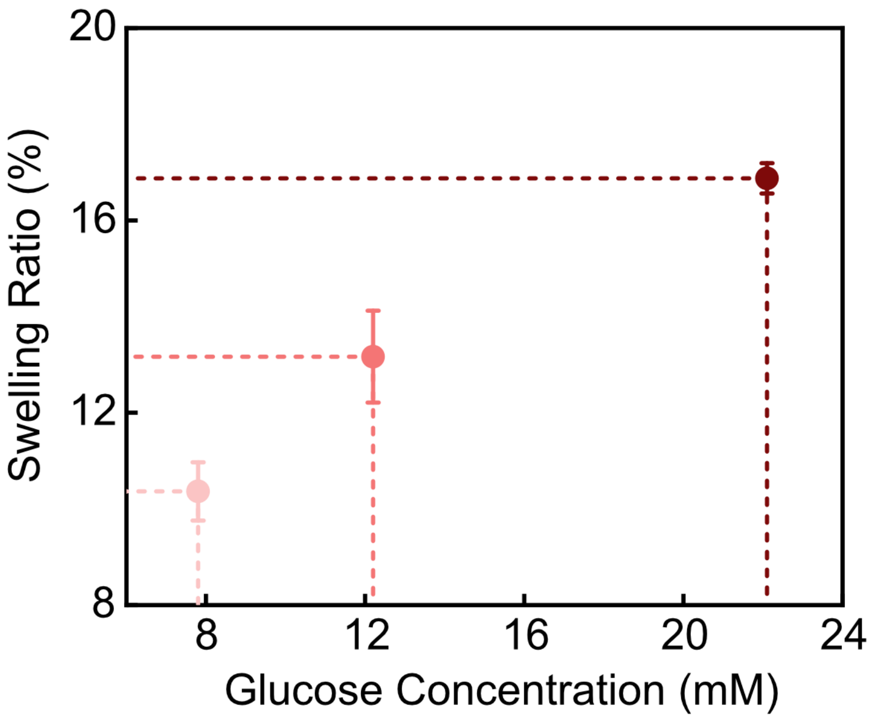

Fig. S14. SRs of the ARMPatches in response to the blood glucose concentration of the mice in vivo obtained via a microscope.

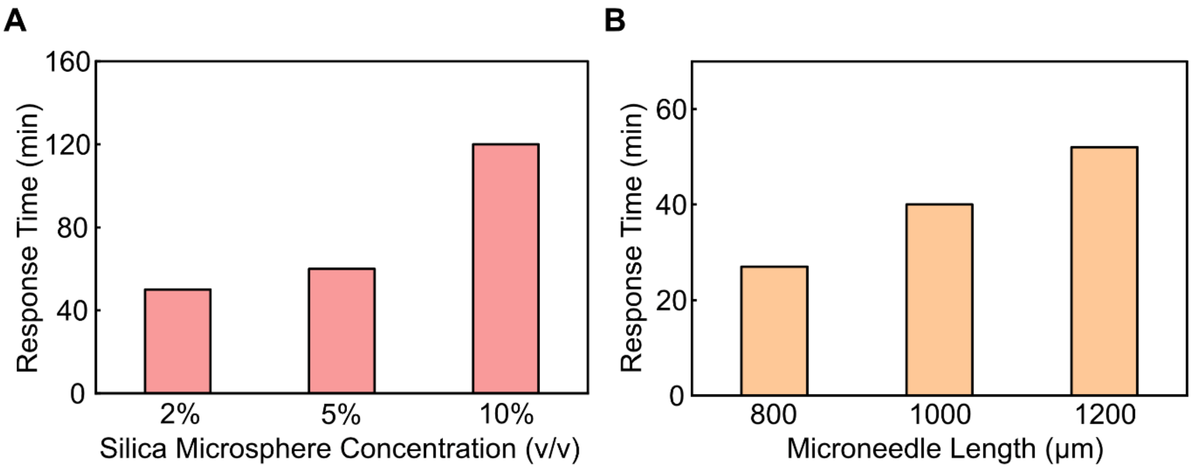

Fig. S15. Optimization of the response time of the ARMPatch. (**A** and **B**) The effect of the hydrogel composition (A) and the microneedle length (B) on the response time of the ARMPatch.

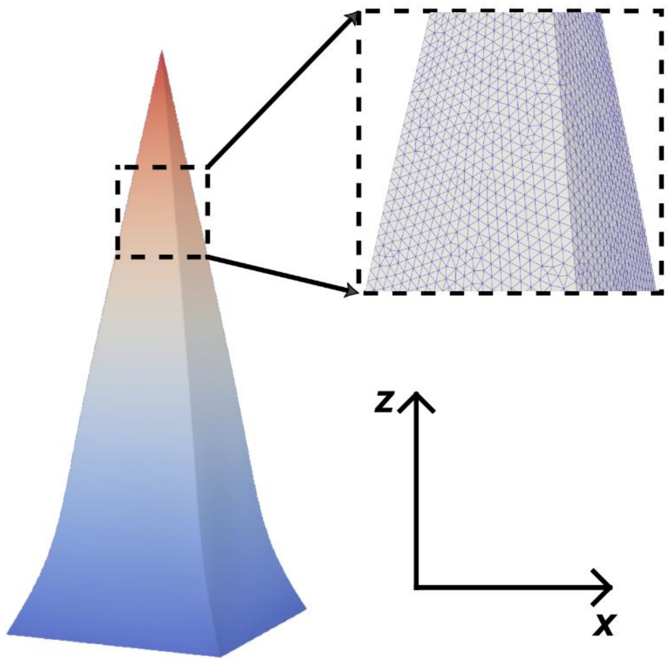

Fig. S16. The geometry of a single microneedle used in the simulation.

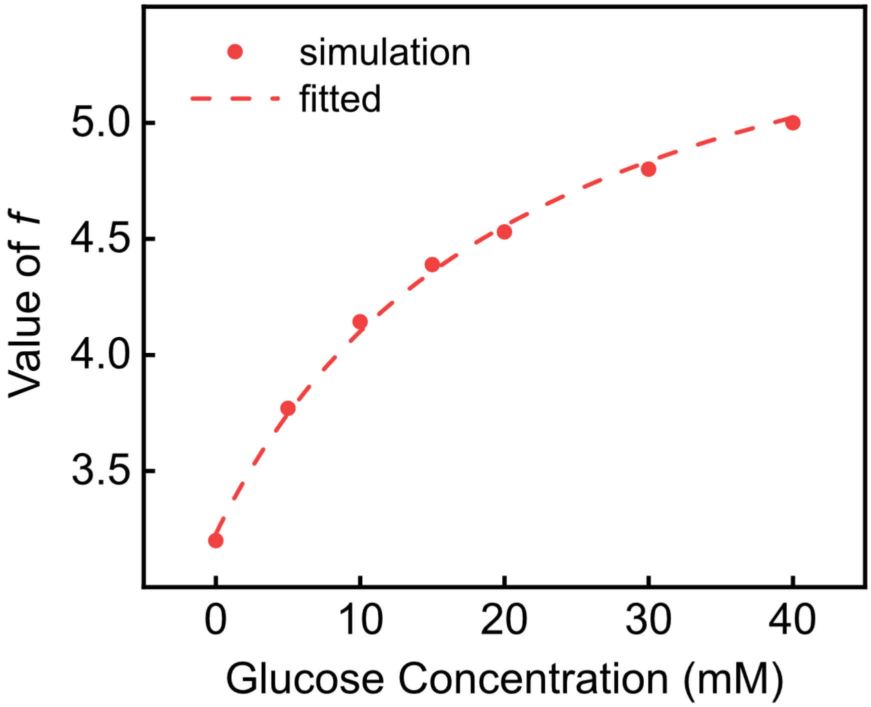

Fig. S17. The comparison of the value of parameter *f* in the simulation as a function of the glucose concentration.

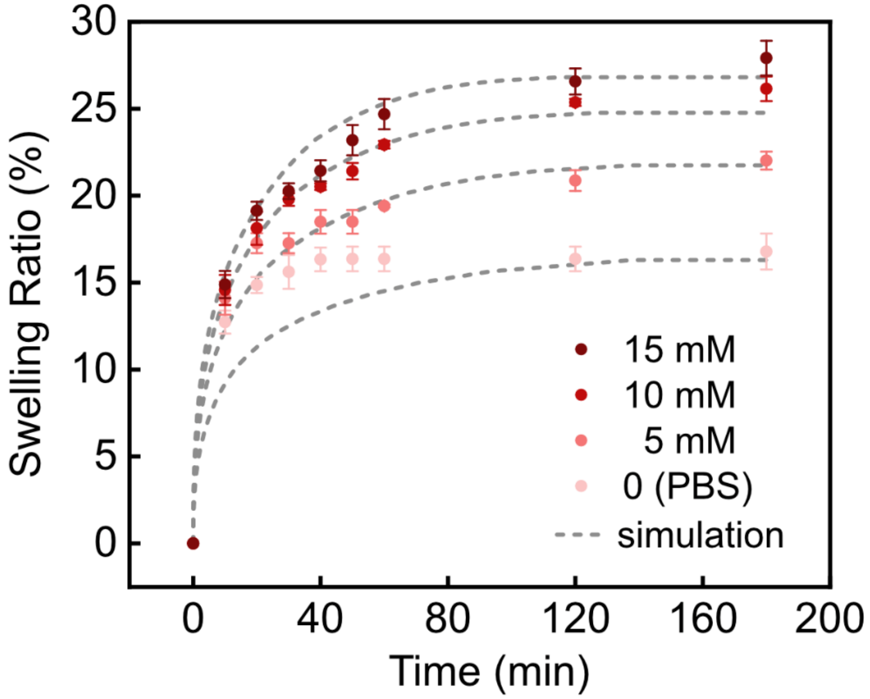

Fig. S18. Comparison of experimental and simulated hydrogel swelling kinetics as a function of time across multiple glucose concentrations.

Table S1 Comparison between the ARMPatch with existing CGM approaches

Techniques	Mechanism	Accuracy	Signal robustness	Stability	Penetration length	Operational usability
This work	**Ultrasound** (hydrogel)	**Medium** (preclinical validation)	**High** (acoustic readout)	**High** (in vivo ~7 days; up to 56 days)	**~ 1 mm** (microneedle)	**High** (ultrasound probe)
Dexcom G7	**Electrochemistry** (enzyme)	**High** (human MARD ~ 8 %)	**High** (algorithm assisted; enzyme dependent)	**Medium** (in vivo < 15 days)	**> 3 mm** (introducer needle)	**Medium** (general electrochemical electronics)
Eversense 365	**Optics** (fluorescence polymer)	**High** (human MARD ~ 9%)	**High** (stable with periodic calibration)	**High** (in vivo < 365 days)	**Invasive** (implantation)	**Low** (customized optical transmitter)
[81]	**Electrochemistry** (nanoparticle)	**High** (detection limit ~5.5 μM)	**Low** (sensitive to pH, temperature)	**Low** (in vivo < 1 day)	**Non-invasive** (sweat sensor)	**Medium** (general electrochemical electronics)
[75]	**Optics** (fluorescence polymer)	**Medium** (preclinical validation)	**Low** (sensitive to ambient light; photobleaching)	**Low** (in vivo < 3 days)	**~1 mm** (microneedle)	**Low** (customized fluorescence light source)
[59]	**Ultrasound** (hydrogel)	**Medium** (preclinical validation)	**High** (acoustic readout)	**Low** (in vivo < 1 day)	**Invasive** (implantation)	**High** (ultrasound probe)
[82]	**OECT** (enzyme)	**High** (detection limit ~1 μM)	**Medium** (dependent on a channel to amplify signals)	**Low** (in vivo < 1 day)	**~1 mm** (microneedle)	**Medium** (general electrochemical electronics)

- MARD: Mean Absolute Relative Difference
(a metric used to measure the accuracy of CGM device by comparing sensor readings to a reference blood glucose measurement)
- OECT: Organic Electrochemical Transistor

Table S2 Leakage elements of hydrogel-soaked water samples

Group	Concentration of elements (mg/L)			
	**B** (by ICP-MS)	**Si** (by ICP-MS)	**C** (by TOC)	**N** (by TOC)
Water sample with the hydrogel	＜0.02 (detection limit)	＜0.02 (detection limit)	＜0.08	＜0.05
Water sample without the hydrogel	＜0.02	＜0.02	＜0.08	＜0.05

Table S3. Fitted parameters in the simulation

Parameter	Value	Unit
Nv^s	0.02	1
f_{tot}	6.0	1
χ	0.2	1
Ka	85.2	L/mol
pKa	7.33	1
D_{h}	1×10^{-10}	m^2/s
D_{l}	1×10^{-11}	m^2/s
J_{mid}	16.5	1
J_{range}	4	1

Movie S1.Dynamic comparison of the ultrasound contrast of the microneedles with and without silica microspheres in ultrasound images via a moving ultrasound probe. Pseudo-color is achieved by modifying the LUT of the movie for visualization.

Movie S2. In vivo dynamic ultrasound imaging of the microneedles of the ARMPatch in a nude mouse model in sync with the heartbeat. Pseudo-color is achieved by modifying the LUT of the movie for visualization.

Movie S3. Video recordings of the tested mouse with normal behaviors over a seven-day period and the healthy state of the mouse after the experiment.

REFERENCES

1. P. Saeedi, P. Salpea, S. Karuranga, I. Petersohn, B. Malanda, E. W. Gregg, N. Unwin, S. H. Wild, R. Williams, Mortality attributable to diabetes in 20–79 years old adults, 2019 estimates: Results from the international diabetes federation diabetes Atlas, 9th edition. *Diabetes Res. Clin. Pract.* **162**, 108086 (2020).

2. J. B. Cole, J. C. Florez, Genetics of diabetes mellitus and diabetes complications. *Nat. Rev. Nephrol.* **16**, 377–390 (2020).

3. S. Pleus, G. Freckmann, S. Schauer, L. Heinemann, R. Ziegler, L. Ji, V. Mohan, L. E. Calliari, R. Hinzmann, Self-monitoring of blood glucose as an integral part in the management of people with type 2 diabetes mellitus. *Diabetes Ther.* **13**, 829–846 (2022).

4. A. Y. Y. Cheng, M. B. Gomes, S. Kalra, A.-P. Kengne, C. Mathieu, J. E. Shaw, Applying the WHO global targets for diabetes mellitus. *Nat. Rev. Endocrinol.* **19**, 194–200 (2023).

5. J. J. Wright, A. J. Williams, S. B. Friedman, R. G. Weaver, J. M. Williams, E. Hodge, M. Fowler, S. Bao, Accuracy of continuous glucose monitors for inpatient diabetes management. *J. Diabetes Sci. Technol.* **17**, 1252–1255 (2023).

6. S. Celik, R. Pınar, Insulin injection and finger sticking fear in diabetic people. *J. Psychiatr. Nurs.* **5**, 104–108 (2014).

7. R. A. Ajjan, T. Battelino, X. Cos, S. Del Prato, J.-C. Philips, L. Meyer, J. Seufert, S. Seidu, Continuous glucose monitoring for the routine care of type 2 diabetes mellitus. *Nat. Rev. Endocrinol.* **20**, 426–440 (2024).

8. I. Lee, D. Probst, D. Klonoff, K. Sode, Continuous glucose monitoring systems—Current status and future perspectives of the flagship technologies in biosensor research. *Biosens. Bioelectron.* **181**, 113054 (2021).

9. B. J. van Enter, E. von Hauff, Challenges and perspectives in continuous glucose monitoring. *Chem. Commun.* **54**, 5032–5045 (2018).

10. H. Teymourian, A. Barfidokht, J. Wang, Electrochemical glucose sensors in diabetes management: An updated review (2010–2020). *Chem. Soc. Rev.* **49**, 7671–7709 (2020).

11. E. González-Martínez, S. K. Saem, N. E. Beganovic, J. M. Moran-Mirabal, Electrochemical nano-roughening of gold microstructured electrodes for enhanced sensing in biofluids. *Angew. Chem. Int. Ed.* **62**, e202218080 (2023).

12. Y. Zhou, Q. Hu, F. Yu, G.-Y. Ran, H.-Y. Wang, N. D. Shepherd, D. M. D'Alessandro, M. Kurmoo, J.-L. Zuo, A metal–organic framework based on a nickel bis(dithiolene) connector: Synthesis, crystal structure, and application as an electrochemical glucose sensor. *J. Am. Chem. Soc.* **142**, 20313–20317 (2020).

13. Q. Zhang, P. Li, J. Wu, Y. Peng, H. Pang, Pyridine-regulated lamellar nickel-based metal–organic framework (Ni-MOF) for nonenzymatic electrochemical glucose sensor. *Adv. Sci.* **10**, e2304102 (2023).

14. M. Ferris, G. Zabow, Quantitative, high-sensitivity measurement of liquid analytes using a smartphone compass. *Nat. Commun.* **15**, 2801 (2024).

15. J. Yang, S. Zheng, D. Ma, T. Zhang, X. Huang, S. Huang, H. Chen, J. Wang, L. Jiang, X. Xie, Masticatory system–inspired microneedle theranostic platform for intelligent and precise diabetic management. *Sci. Adv.* **8**, eabo6900 (2022).

16. W. He, C. Wang, H. Wang, M. Jian, W. Lu, X. Liang, X. Zhang, F. Yang, Y. Zhang, Integrated textile sensor patch for real-time and multiplex sweat analysis. *Sci. Adv.* **5**, eaax0649 (2019).

17. N. A. Peppas, J. Z. Hilt, A. Khademhosseini, R. Langer, Hydrogels in biology and medicine: From molecular principles to bionanotechnology. *Adv. Mater.* **18**, 1345–1360 (2006).

18. C. Zhang, M. D. Losego, P. V. Braun, Hydrogel-based glucose sensors: Effects of phenylboronic acid chemical structure on response. *Chem. Mater.* **25**, 3239–3250 (2013).

19. R. V. Ulijn, N. Bibi, V. Jayawarna, P. D. Thornton, S. J. Todd, R. J. Mart, A. M. Smith, J. E. Gough, Bioresponsive hydrogels. *Mater. Today* **10**, 40–48 (2007).

20. N. Gao, H. You, Recent applications of point-of-care devices for glucose detection on the basis of stimuli-responsive volume phase transition of hydrogel. *Biochip J.* **15**, 23–41 (2021).

21. X. Zhao, X. Chen, H. Yuk, S. Lin, X. Liu, G. Parada, Soft materials by design: Unconventional polymer networks give extreme properties. *Chem. Rev.* **121**, 4309–4372 (2021).

22. Y. Guan, H. Niu, Z. Liu, Y. Dang, J. Shen, M. Zayed, L. Ma, J. Guan, Sustained oxygenation accelerates diabetic wound healing by promoting epithelialization and angiogenesis and decreasing inflammation. *Sci. Adv.* **7**, eabj0153 (2021).

23. J. Song, Y. Zhang, S. Y. Chan, Z. Du, Y. Yan, T. Wang, P. Li, W. Huang, Hydrogel-based flexible materials for diabetes diagnosis, treatment, and management. *NPJ Flexible Electron.* **5**, 1–17 (2021).

24. P. Makvandi, R. Jamaledin, G. Chen, Z. Baghbantaraghdari, E. N. Zare, C. Di Natale, V. Onesto, R. Vecchione, J. Lee, F. R. Tay, P. Netti, V. Mattoli, A. Jaklenec, Z. Gu, R. Langer, Stimuli-responsive transdermal microneedle patches. *Mater. Today* **47**, 206–222 (2021).

25. J. Wang, Z. Wang, J. Yu, A. R. Kahkoska, J. B. Buse, Z. Gu, Glucose-responsive insulin and delivery systems: Innovation and translation. *Adv. Mater.* **32**, e1902004 (2020).

26. A. GhavamiNejad, J. F. Liu, S. Mirzaie, B. Lu, M. Samarikhalaj, A. Giacca, X. Y. Wu, Catechol-based chemistry for hypoglycemia-responsive delivery of zinc-glucagon via hydrogel-based microneedle patch technology. *Nat. Commun.* **16**, 3124 (2025).

27. V. L. Alexeev, A. C. Sharma, A. V. Goponenko, S. Das, I. K. Lednev, C. S. Wilcox, D. N. Finegold, S. A. Asher, High ionic strength glucose-sensing photonic crystal. *Anal. Chem.* **75**, 2316–2323 (2003).

28. Y. J. Heo, H. Shibata, T. Okitsu, T. Kawanishi, S. Takeuchi, Long-term in vivo glucose monitoring using fluorescent hydrogel fibers. *Proc. Natl. Acad. Sci. U.S.A.* **108**, 13399–13403 (2011).

29. A. K. Yetisen, N. Jiang, A. Fallahi, Y. Montelongo, G. U. Ruiz-Esparza, A. Tamayol, Y. S. Zhang, I. Mahmood, S.-A. Yang, K. S. Kim, H. Butt, A. Khademhosseini, S.-H. Yun, Glucose-sensitive hydrogel optical fibers functionalized with phenylboronic acid. *Adv. Mater.* **29**, 1606380 (2017).

30. H. Shibata, Y. J. Heo, T. Okitsu, Y. Matsunaga, T. Kawanishi, S. Takeuchi, Injectable hydrogel microbeads for fluorescence-based in vivo continuous glucose monitoring. *Proc. Natl. Acad. Sci. U.S.A.* **107**, 17894–17898 (2010).

31. M. Ben-Moshe, V. L. Alexeev, S. A. Asher, Fast responsive crystalline colloidal array photonic crystal glucose sensors. *Anal. Chem.* **78**, 5149–5157 (2006).

32. M. Elsherif, M. U. Hassan, A. K. Yetisen, H. Butt, Wearable contact lens biosensors for continuous glucose monitoring using smartphones. *ACS Nano* **12**, 5452–5462 (2018).

33. Y. Zeng, J. Wang, Z. Wang, G. Chen, J. Yu, S. Li, Q. Li, H. Li, D. Wen, Z. Gu, Z. Gu, Colloidal crystal microneedle patch for glucose monitoring. *Nano Today* **35**, 100984 (2020).

34. Z. Yu, N. Jiang, S. G. Kazarian, S. Tasoglu, A. K. Yetisen, Optical sensors for continuous glucose monitoring. *Prog. Biomed. Eng.* **3**, 022004 (2021).

35. M. Adeel, Md. M. Rahman, I. Caligiuri, V. Canzonieri, F. Rizzolio, S. Daniele, Recent advances of electrochemical and optical enzyme-free glucose sensors operating at physiological conditions. *Biosens. Bioelectron.* **165**, 112331 (2020).

36. G. P. C. Mello, E. F. C. Simões, D. M. A. Crista, J. M. M. Leitão, L. Pinto da Silva, J. C. G. Esteves da Silva, Glucose sensing by fluorescent nanomaterials. *Crit. Rev. Anal. Chem.* **49**, 542–552 (2019).

37. R. Alford, H. M. Simpson, J. Duberman, G. C. Hill, M. Ogawa, C. Regino, H. Kobayashi, P. L. Choyke, Toxicity of organic fluorophores used in molecular imaging: Literature review. *Mol. Imaging* **8**, 341–354 (2009).

38. D. C. Klonoff, Overview of fluorescence glucose sensing: A technology with a bright future. *J. Diabetes Sci. Technol.* **6**, 1242–1250 (2012).

39. M. Elsherif, F. Alam, A. E. Salih, B. AlQattan, A. K. Yetisen, H. Butt, Wearable bifocal contact lens for continual glucose monitoring integrated with smartphone readers. *Small* **17**, e2102876 (2021).

40. A. K. Yetisen, Y. Montelongo, F. da Cruz Vasconcellos, J. L. Martinez-Hurtado, S. Neupane, H. Butt, M. M. Qasim, J. Blyth, K. Burling, J. B. Carmody, M. Evans, T. D. Wilkinson, L. T. Kubota, M. J. Monteiro, C. R. Lowe, Reusable, robust, and accurate laser-generated photonic nanosensor. *Nano Lett.* **14**, 3587–3593 (2014).

41. Y. Jing, S. J. Chang, C.-J. Chen, J.-T. Liu, Review—Glucose monitoring sensors: History, principle, and challenges. *J. Electrochem. Soc.* **169**, 057514 (2022).

42. G. ter Haar, Ultrasound bioeffects and safety. *Proc. Inst. Mech. Eng. H* **224**, 363–373 (2010).

43. M. Natori, Ultrasound safety: Overview and what we do need in daily clinics for a safe use of diagnostic ultrasound. *Int. Congr. Ser.* **1274**, 125–128 (2004).

44. B. Shin, S. Jeon, J. Ryu, H. J. Kwon, Elastography for portable ultrasound. *Biomed. Eng. Lett.* **8**, 101–116 (2018).

45. H. Hu, H. Huang, M. Li, X. Gao, L. Yin, R. Qi, R. S. Wu, X. Chen, Y. Ma, K. Shi, C. Li, T. M. Maus, B. Huang, C. Lu, M. Lin, S. Zhou, Z. Lou, Y. Gu, Y. Chen, Y. Lei, X. Wang, R. Wang, W. Yue, X. Yang, Y. Bian, J. Mu, G. Park, S. Xiang, S. Cai, P. W. Corey, J. Wang, S. Xu, A wearable cardiac ultrasound imager. *Nature* **613**, 667–675 (2023).

46. S. Zhou, G. Park, K. Longardner, M. Lin, B. Qi, X. Yang, X. Gao, H. Huang, X. Chen, Y. Bian, H. Hu, R. S. Wu, W. Yue, M. Li, C. Lu, R. Wang, S. Qin, E. Tasali, T. Karrison, I. Thomas, B. Smarr, E. B. Kistler, B. A. Khiami, I. Litvan, S. Xu, Clinical validation of a wearable ultrasound sensor of blood pressure. *Nat. Biomed. Eng.* **9**, 865–881 (2025).

47. C. Wang, X. Chen, L. Wang, M. Makihata, H.-C. Liu, T. Zhou, X. Zhao, Bioadhesive ultrasound for long-term continuous imaging of diverse organs. *Science* **377**, 517–523 (2022).

48. M. T. Chorsi, T. T. Le, F. Lin, T. Vinikoor, R. Das, J. F. Stevens, C. Mundrane, J. Park, K. T. M. Tran, Y. Liu, J. Pfund, R. Thompson, W. He, M. Jain, M. D. Morales-Acosta, O. R. Bilal, K. Kazerounian, H. Ilies, T. D. Nguyen, Highly piezoelectric, biodegradable, and flexible amino acid nanofibers for medical applications. *Sci. Adv.* **9**, eadg6075 (2023).

49. J. Yuan, Z. Li, Y. Zhao, R. Luo, S. Qin, J. Li, M. Li, G. Han, Z. Li, Z. Zhao, J. Li, S. Zhang, Z. Yuan, X. Han, L. Fan, X. Wang, T. Wang, P. Yang, L. Zhao, L. Yuan, Y. Lv, R. Wu, T. K. Hsiai, Z. Jiang, Skin-adaptive focused flexible micromachined ultrasound transducers for wearable cardiovascular health monitoring. *Sci. Adv.* **11**, eadw7632 (2025).

50. F. Wang, P. Jin, Y. Feng, J. Fu, P. Wang, X. Liu, Y. Zhang, Y. Ma, Y. Yang, A. Yang, X. Feng, Flexible Doppler ultrasound device for the monitoring of blood flow velocity. *Sci. Adv.* **7**, eabi9283 (2021).

51. H.-C. Liu, Y. Zeng, C. Gong, X. Chen, P. Kijanka, J. Zhang, Y. Genyk, H. Tchelepi, C. Wang, Q. Zhou, X. Zhao, Wearable bioadhesive ultrasound shear wave elastography. *Sci. Adv.* **10**, eadk8426 (2024).

52. C. Li, H. Wang, Z. Song, W. Zhang, Y. Pan, Z. Zhao, C. Qiu, K. Yin, M. Han, A. B. Wang, H. Luan, J. Li, W. Yan, S. Chen, H. Shen, T.-L. Liu, S. S. M. Lee, W. Ding, Y. Huang, J. A. Rogers, C. Wu, X. Ni, Wireless, wearable elastography via mechano-acoustic wave sensing for ambulatory monitoring of tissue stiffness. *Sci. Adv.* **11**, eady0534 (2025).

53. F. Zou, Y. Liu, Y. Luo, T. Xu, A wearable spatiotemporal controllable ultrasonic device with amyloid-β disaggregation for continuous Alzheimer's disease therapy. *Sci. Adv.* **11**, eadw1732 (2025).

54. W. Du, L. Zhang, E. Suh, D. Lin, C. Marcus, L. Ozkan, A. Ahuja, S. Fernandez, I. I. Shuvo, D. Sadat, W. Liu, F. Li, A. P. Chandrakasan, T. Ozmen, C. Dagdeviren, Conformable ultrasound breast patch for deep tissue scanning and imaging. *Sci. Adv.* **9**, eadh5325 (2023).

55. J. Liu, N. Liu, Y. Xu, M. Wu, H. Zhang, Y. Wang, Y. Yan, A. Hill, R. Song, Z. Xu, M. Park, Y. Wu, J. L. Ciatti, J. Gu, H. Luan, Y. Zhang, T. Yang, H.-Y. Ahn, S. Li, W. Z. Ray, C. K. Franz, M. R. MacEwan, Y. Huang, C. W. Hammill, H. Wang, J. A. Rogers, Bioresorbable shape-adaptive structures for ultrasonic monitoring of deep-tissue homeostasis. *Science* **383**, 1096–1103 (2024).

56. H. Tang, Y. Yang, Z. Liu, W. Li, Y. Zhang, Y. Huang, T. Kang, Y. Yu, N. Li, Y. Tian, X. Liu, Y. Cheng, Z. Yin, X. Jiang, X. Chen, J. Zang, Injectable ultrasonic sensor for wireless monitoring of intracranial signals. *Nature* **630**, 84–90 (2024).

57. J. H. Park, A. Kim, H. Jiang, S. H. Song, J. Zhou, B. Ziaie, A wireless chemical sensing scheme using ultrasonic imaging of silica-particle-embedded hydrogels (Silicagel). *Sens. Actuators B Chem.* **259**, 552–559 (2018).

58. H. Jiang, N. M. Carter, A. Zareei, S. Nejati, J. F. Waimin, S. Chittiboyina, E. E. Niedert, T. Soleimani, S. A. Lelièvre, C. J. Goergen, R. Rahimi, A wireless implantable strain sensing scheme using ultrasound imaging of highly stretchable zinc oxide/poly dimethylacrylamide nanocomposite hydrogel. *ACS Appl. Bio Mater.* **3**, 4012–4024 (2020).

59. N. Farhoudi, L. B. Laurentius, J. J. Magda, C. F. Reiche, F. Solzbacher, In vivo monitoring of glucose using ultrasound-induced resonance in implantable smart hydrogel microstructures. *ACS Sens.* **6**, 3587–3595 (2021).

60. D. Troïani, J. R. Dion, D. H. Burns, Ultrasonic quantification using smart hydrogel sensors. *Talanta* **83**, 1371–1375 (2011).

61. M. Ghosh, V. R. Bora, Evolution in blood glucose monitoring: A comprehensive review of invasive to non-invasive devices and sensors. *Discov. Med.* **2**, 74 (2025).

62. L. Braunstein, S. C. Brüningk, I. Rivens, J. Civale, G. ter Haar, Characterization of acoustic, cavitation, and thermal properties of poly(vinyl alcohol) hydrogels for use as therapeutic ultrasound tissue mimics. *Ultrasound Med. Biol.* **48**, 1095–1109 (2022).

63. E. De la Paz, A. Barfidokht, S. Rios, C. Brown, E. Chao, J. Wang, Extended noninvasive glucose monitoring in the interstitial fluid using an epidermal biosensing patch. *Anal. Chem.* **93**, 12767–12775 (2021).

64. F. Tehrani, H. Teymourian, B. Wuerstle, J. Kavner, R. Patel, A. Furmidge, R. Aghavali, H. Hosseini-Toudeshki, C. Brown, F. Zhang, K. Mahato, Z. Li, A. Barfidokht, L. Yin, P. Warren, N. Huang, Z. Patel, P. P. Mercier, J. Wang, An integrated wearable microneedle array for the continuous monitoring of multiple biomarkers in interstitial fluid. *Nat. Biomed. Eng.* **6**, 1214–1224 (2022).

65. Z. Wang, R. Fu, X. Han, D. Wen, Y. Wu, S. Li, Z. Gu, Shrinking fabrication of a glucose-responsive glucagon microneedle patch. *Adv. Sci.* **9**, e2203274 (2022).

66. Z. Wang, H. Li, J. Wang, Z. Chen, G. Chen, D. Wen, A. Chan, Z. Gu, Transdermal colorimetric patch for hyperglycemia sensing in diabetic mice. *Biomaterials* **237**, 119782 (2020).

67. J. Yu, J. Wang, Y. Zhang, G. Chen, W. Mao, Y. Ye, A. R. Kahkoska, J. B. Buse, R. Langer, Z. Gu, Glucose-responsive insulin patch for the regulation of blood glucose in mice and minipigs. *Nat. Biomed. Eng.* **4**, 499–506 (2020).

68. S. P. Davis, B. J. Landis, Z. H. Adams, M. G. Allen, M. R. Prausnitz, Insertion of microneedles into skin: Measurement and prediction of insertion force and needle fracture force. *J. Biomech.* **37**, 1155–1163 (2004).

69. L. Monnier, C. Colette, D. Owens, Postprandial and basal glucose in type 2 diabetes: Assessment and respective impacts. *Diabetes Technol. Ther.* **13**, S-25–S-32 (2011).

70. J.-S. Wang, S.-T. Tu, I.-T. Lee, S.-D. Lin, S.-Y. Lin, S.-L. Su, W.-J. Lee, W. H.-H. Sheu, Contribution of postprandial glucose to excess hyperglycaemia in Asian type 2 diabetic patients using continuous glucose monitoring. *Diabetes Metab. Res. Rev.* **27**, 79–84 (2011).

71. S. Zoet, T. Urgert, A. Veldhuis, B.-J. van Beijnum, G. D. Laverman, Quantification of the relation between continuous glucose monitoring observation period and the estimation error in assessing long-term glucose regulation. *BMJ Open Diabetes Res. Care* **13**, e004768 (2025).

72. S. M. Alexanian, M. C. Cheney, J. C. Bello Ramos, N. L. Spartano, H. A. Wolpert, D. W. Steenkamp, Impact of meal insulin bolus timing and bedtime snacking on continuous glucose monitoring–derived glycemic metrics in hospitalized inpatients. *Diabetes Technol. Ther.* **27**, 511–516 (2025).

73. W. Hong, X. Zhao, J. Zhou, Z. Suo, A theory of coupled diffusion and large deformation in polymeric gels. *J. Mech. Phys. Solids* **56**, 1779–1793 (2008).

74. W. Hong, X. Zhao, Z. Suo, Large deformation and electrochemistry of polyelectrolyte gels. *J. Mech. Phys. Solids* **58**, 558–577 (2010).

75. M. Sang, M. Cho, S. Lim, I. S. Min, Y. Han, C. Lee, J. Shin, K. Yoon, W.-H. Yeo, T. Lee, S. M. Won, Y. Jung, Y. J. Heo, K. J. Yu, Fluorescent-based biodegradable microneedle sensor array for tether-free continuous glucose monitoring with smartphone application. *Sci. Adv.* **9**, eadh1765 (2023).

76. J. Zhang, Y. Zheng, J. Lee, A. Hoover, S. A. King, L. Chen, J. Zhao, Q. Lin, C. Yu, L. Zhu, X. Wu, Continuous glucose monitoring enabled by fluorescent nanodiamond boronic hydrogel. *Adv. Sci.* **10**, e2203943 (2023).

77. H. Xue, J. Jin, X. Huang, Z. Tan, Y. Zeng, G. Lu, X. Hu, K. Chen, Y. Su, X. Hu, X. Peng, L. Jiang, J. Wu, Wearable flexible ultrasound microneedle patch for cancer immunotherapy. *Nat. Commun.* **16**, 2650 (2025).

78. H. Kim, J. Lee, U. Heo, D. K. Jayashankar, K.-C. Agno, Y. Kim, C. Y. Kim, Y. Oh, S.-H. Byun, B. Choi, H. Jeong, W.-H. Yeo, Z. Li, S. Park, J. Xiao, J. Kim, J.-W. Jeong, Skin preparation-free, stretchable microneedle adhesive patches for reliable electrophysiological sensing and exoskeleton robot control. *Sci. Adv.* **10**, eadk5260 (2024).

79. C. Permann, D. Gaston, D. Andrš, R. Carlsen, F. Kong, A. Lindsay, J. Miller, J. Peterson, A. Slaughter, R. Stogner, R. Martineau, MOOSE: Enabling massively parallel multiphysics simulations. *SoftwareX* **11**, 100430 (2020).

80. J. Yan, G. Springsteen, S. Deeter, B. Wang, The relationship among pKa, pH, and binding constants in the interactions between boronic acids and diols—It is not as simple as it appears. *Tetrahedron* **60**, 11205–11209 (2004).

81. W. Huang, Y. Yang, Y. Xu, F. Xiao, L. Wang, Sweat wearable sensor based on confined Pt nanoparticles in 2D conductive metal–organic frameworks for continuous glucose monitoring. *Adv. Sci.* **35**, e07212 (2025).

82. J. Bai, D. Liu, X. Tian, Y. Wang, B. Cui, Y. Yang, S. Dai, W. Lin, J. Zhu, J. Wang, A. Xu, Z. Gu, S. Zhang, Coin-sized, fully integrated, and minimally invasive continuous glucose monitoring system based on organic electrochemical transistors. *Sci. Adv.* **10**, eadl1856 (2024).
